# Membrane permeabilizing amphiphilic peptide delivers recombinant transcription factor and CRISPR-Cas9/Cpf1 ribonucleoproteins in hard-to-modify cells

**DOI:** 10.1371/journal.pone.0195558

**Published:** 2018-04-04

**Authors:** Thomas Del’Guidice, Jean-Pascal Lepetit-Stoffaes, Louis-Jean Bordeleau, Joannie Roberge, Vanessa Théberge, Coraline Lauvaux, Xavier Barbeau, Jessica Trottier, Vibhuti Dave, Denis-Claude Roy, Bruno Gaillet, Alain Garnier, David Guay

**Affiliations:** 1 Feldan Therapeutics, Québec, Québec, Canada; 2 Université Laval, Département de Génie Chimique, Québec, Québec, Canada; 3 Centre de recherche de l’Hôpital Maisonneuve-Rosemont, Montréal, Québec, Canada; University of Florida, UNITED STATES

## Abstract

Delivery of recombinant proteins to therapeutic cells is limited by a lack of efficient methods. This hinders the use of transcription factors or Clustered Regularly Interspaced Short Palindromic Repeats (CRISPR) ribonucleoproteins to develop cell therapies. Here, we report a soluble peptide designed for the direct delivery of proteins to mammalian cells including human stem cells, hard-to-modify primary natural killer (NK) cells, and cancer cell models. This peptide is composed of a 6x histidine-rich domain fused to the endosomolytic peptide CM18 and the cell penetrating peptide PTD4. A less than two-minute co-incubation of 6His-CM18-PTD4 peptide with spCas9 and/or asCpf1 CRISPR ribonucleoproteins achieves robust gene editing. The same procedure, co-incubating with the transcription factor HoxB4, achieves transcriptional regulation. The broad applicability and flexibility of this DNA- and chemical-free method across different cell types, particularly hard-to-transfect cells, opens the way for a direct use of proteins for biomedical research and cell therapy manufacturing.

## Introduction

Current viral and non-viral intracellular transfer methods mainly rely on the *ex vivo* delivery of foreign genetic material. These methods are currently limited by regulatory, safety and economic hurdles to cell therapy development [1–3]. The direct delivery of recombinant proteins to cells is a promising approach to overcome these impediments [4–6]. Unfortunately, this avenue as yet suffers from the scarcity of safe and efficient protein delivery methods [7, 8].

Fusion of recombinant proteins to cell-penetrating peptides (CPPs), which are cationic peptides with the ability to bind and translocate though cell membranes, promote the delivery of various molecular cargos to living cells. However, CPP-mediated protein delivery usually results in low cytosolic distribution due to massive sequestration of cargoes in endosomes [9–12].

A strategy to avoid this endosomal entrapment is to combine a CPP with endosomal leakage domains (ELDs), which are endosomolytic peptides that bind and destabilize endosomal membranes [13, 14]. The fusion of CPPs to ELDs is an emerging method; proofs of concept have required the use of toxic fluorescent dye, long incubation time, or organic solvents to solubilize the peptide [15–18]. Now that the CPP-ELD delivery system has been shown to work, research can advance to producing chemical-free and water-soluble peptides with high protein delivery efficiency to multiple cell types. This technology should have a high potential utility in therapeutic applications and cell manufacturing.

Here we report a peptide comprising both a CPP and an ELD that mediates the rapid, safe, and efficient cytosolic delivery of functional proteins to 20 mammalian cell types, including human stem cells, human primary cells and cancer cell models. To achieve these delivery results, we fused the CPP Human Immunodeficiency Virus (HIV)-TAT variant PTD4 (YARAAARQARA) [19], with the cationic amphiphilic α-helical ELD CM18 (KWKLFKKIGAVLKVLTTG) [13]. We geared this chimeric peptide with six consecutive histidine residues in the N-terminal position to improve endosomal destabilization through their proton sponge effect [20, 21]. Importantly, the 6His-CM18-PTD4 peptide was not fused or conjugated to protein cargoes.

This method was optimal to easily adjust concentrations and cell incubation time while maintaining the protein’s structural integrity. Functional transcription factor HoxB4, CRISPR Cas9, and Cpf1 ribonucleoprotein (RNP) complexes were delivered to living cells using a simple co-incubation protocol. Protein uptake could be performed in less than 2 minutes or maintained for hours and days. This soluble, chemical-free and easy-to-use peptide opens new avenues to expand, differentiate, reprogram or edit therapeutic cells by the use of transcription factors, CRISPR RNP or any recombinant proteins.

## Materials and methods

Detailed methods are provided in the online version of this paper and include the list of peptides, proteins, cell types, gene targets, guide RNAs, size of cleavage products and secondary structure prediction of peptides provided in S1 to S6 Tables, respectively.

### Contact for reagents and resource sharing

Further information and requests for resources and reagents should be directed to and will be fulfilled by the Lead Contact, David Guay (mailto:dguay@feldan.com). Cell cultures, peptides and reagents were purchased from commercial companies. Proteins were manufactured by the Feldan Therapeutics research department.

### Experimental methods

#### Cell culture

HeLa, HEK293T, THP-1, Jurkat, CHO, NIH3T3, CA46, Balb3T3, HT2, KMS-12, DOHH2, REC-1, HCC 78 and NCI-H196 cells were obtained from American Type Culture Collection (Manassas, VA, USA) and cultured following the manufacturer's instructions. CD34+ cells were cultured and used for cell delivery purpose at CETC-Hôpital Rosemont (Montreal, QC, Canada). MSCs, ESCs and iPSCs were cultured and used for cell delivery purpose at CCRM (Toronto, ON, CAN). Myoblasts are primary human cells kindly provided by Professor J.P. Tremblay (Université Laval, Quebec, QC, Canada). Normal Peripheral Blood CD56+ NK cells are natural killer cells obtained from Allcells Inc. (Alameda, CA, USA). Cortical cells are primary cells extracted from rat brain and cultured and used for cell delivery purpose at NIH (Bethesda, MD, USA).

#### Control of gene expression by intracellularly delivered HoxB4 with the 6His-CM18-PTD4 peptide in living cells

THP-1 cells were plated at 1 x 10^6^ cells/well in 800 μL one day before transduction in a 24 well-plate. The next day, in separate sterile 1.5-mL tubes, the peptide was diluted in sterile distilled water at room temperature. HoxB4 protein was mixed with the peptide in cell culture medium with serum in a sufficient final volume for incubation with cells (e.g., 50 μL per well in a 24-well plate). Cells were incubated with the mix for predetermined time exposure at 37°C. Then, cells were centrifuged for 2 min at 400 g, the medium was then removed and the cells were resuspended in PBS previously warmed at 37°C. The cells were subjected to real time-PCR to confirm HoxB4 activity via the measure of the mRNA synthesis levels of HoxB4-targeted gene. HoxB4 activity was normalized to the target gene mRNA levels detected in the negative control cells (no treatment), to obtain a “Fold over control” value. Total RNA levels (ng/μL) were also measured as a marker for cell viability.

#### Genome editing after intracellular delivery of CRISPR systems by the 6His-CM18-PTD4 peptide

HeLa cells were plated at 10000 cells/well one day before transduction. Jurkat and NK cells were plated at 50000 cells/well one day before transduction. The next day, in separate sterile 1.5-mL tubes, 6His-CM18-PTD4 (20 μM) was diluted in sterile distilled water at room temperature (if the cargo is or comprised a nucleic acid, nuclease-free water was used). Cas9-NLS protein (2.5 μM) was mixed with a tracrRNA—crRNA duplex (2 μM), and Cpf1-NLS (1.33 μM) was mixed with crRNA (2 μM), for 5 min in PBS at RT for complexation targeting a nucleotide sequence of the PPIB, HPRT, B2M or DNMT3B genes. Then, CRISPR/Cas9 RNP complex was added to His-CM18-PTD4 (10 μM) and PBS was added to obtain the desired concentration of His-CM18-PTD4 and CRISPR/Cas9 or CRISPR/Cpf1 RNP complex in a sufficient final volume (e.g., 100 μL per well in a 96-well plate). Adherent hela cells were washed with warmed PBS (37°C) just before directly adding the mix for 2 min at RT. Cells in suspension were centrifuged (400 g for 2 min), washed with warmed PBS (37°C) and then centrifuged (400 g for 2 min) before pellet was suspended with the mix for 2 min at RT. Immediately after 2 min, warmed medium with serum (37°) was added in adherent and suspension cells followed by one wash step with PBS. Cells were incubated in medium with serum at 37°C for 24 h or 48 h before RNA extraction and T7E1 assay.

#### Protein purification and fraction

Fusion proteins were expressed in bacteria (*E*. *coli* BL21DE3) under standard conditions using an isopropyl β-D-1-thiogalactopyranoside (IPTG) inducible vector containing a T5 promoter. Solubilized proteins were loaded, using a FPLC (AKTA Explorer 100R), on HisTrapTM FF column previously equilibrated with 5 column volumes (CV) of Tris buffer. Proteins were eluted with 5 CV of Tris buffer with 350 mM Imidazole and collected. Collected fractions corresponding to specific proteins were determined by standard denaturing SDS-PAGE. Culture media contained 24 g yeast extract, 12 g tryptone, 4 mL glycerol, 2.3 g KH2PO4, and 12.5 g K2HPO4 per liter. Bacterial broth was incubated at 37°C under agitation with appropriate antibiotic (e.g., ampicillin). Expression was induced at optical density (600 nm) between 0.5 and 0.6 with a final concentration of 1 mM IPTG for 3 h at 30°C. Bacteria were recuperated following centrifugation at 5000 RPM and bacterial pellets were stored at -20°C. Bacterial pellets were resuspended in Tris buffer (Tris 25 mM pH 7.5, NaCl 100mM, imidazole 5 mM) with phenylmethylsulfonyl fluoride (PMSF) 1 mM, and lysed by passing 3 times through the homogenizer Panda 2KTM at 1000 bar. The solution was centrifuged at 15000 RPM, 4°C for 30 minutes. Supernatants were collected and filtered with a 0.22 μM filtration device. The column was washed with 30 column volumes (CV) of Tris buffer and followed with 30 CV of Tris buffer with imidazole 40 mM. In the case if endotoxin level was still too high considering clinically acceptable criteria (< 100 EU/mg), soluble recombinant proteins were next loaded on Heparin column (20 ml FF—5–10°C) and washed with 20 CV of NaCl (2M) before elution. Purified proteins were diluted in Tris 20 mM at the desired pH according to the protein’s pI and loaded on an appropriate ion exchange column (Q SepharoseTM or SP SepharoseTM) previously equilibrated with 5 CV of Tris 20 mM, NaCl 30 mM. The column was washed with 10 CV of Tris 20 mM, NaCl 30 mM and proteins were eluted with a NaCl gradient until 1 M on 15 CV. Collected fractions corresponding to specific proteins were determined by standard denaturing SDS-PAGE. Purified proteins were then washed and concentrated in PBS 1X on Amicon UltraTM centrifugal filters 10,000 MWCO. Protein concentration was evaluated using a standard Bradford assay.

#### Protein transduction protocol

One day before the transduction assay was performed, cells in exponential growth phase were harvested and plated in a 96-well plate (20,000 cells per well). The cells were incubated overnight in appropriate growth media containing serum. The next day, in separate sterile 1.5-mL tubes, peptide was diluted in sterile distilled water at room temperature (if the cargo is or comprised a nucleic acid, nuclease-free water was used). Protein or RNP was then added to the peptide and, if necessary, sterile PBS or cell culture medium (serum-free) was added to obtain the desired concentrations of shuttle agent and protein in a sufficient final volume to resuspend the cells for one minute (e.g., 10 to 100 μL per well in a 96-well plate). For adherent cells, the media in wells was removed, cells were washed once with PBS previously warmed at 37°C, and the cells were incubated with the cargo protein/peptide mixture at 37°C for one minute. The peptide/cargo mixture in wells was removed, the cells were washed once with PBS, and fresh complete medium was added. Before analysis, the cells were washed once with PBS one last time and fresh complete medium was added. Cells in suspension were centrifuged for 2 min at 400 g and washed with warmed PBS1X before to be resuspended with the cargo protein/peptide mixture at 37°C for the desired length of time. After that, 200 μL of complete medium was added directly on the cells. Cells were centrifuged for 2 min at 400 g and washed with warmed PBS1X before to be resuspended in 50 μL of trypsin-EDTA solution diluted in PBS (1/10) for 2 min. After trypsin inactivation with complete medium, cells were centrifuged for 2 min at 400 g and washed with warmed PBS1X before to be resuspended in 200 μL of complete medium.

#### Lipid transfection assays

CRISPRMax lipofectamine reagent was used following the manufacturer protocol recommendations (Thermofisher).

#### Endotoxin LAL assay

Purified recombinant protein were diluted in water and vortexed for 1 min several times. Limule amoebocyte lysate (BioLynx Inc. #SKC00315) and steril endotoxin standard control solution (*E*.*Coli* 0113:H10) (BioLynx Inc. #SKE0005) were used to measured endoxin levels by spectrophotometry.

#### Synthetic peptides and nucleic acid sequences

CM18 and PTD4 peptides and variants used in this study was purchased from GLBiochem (Shanghai, China). All synthetic RNAs were purchased from Dharmacon (Colorada, US), Millipore Sigma-Aldrich (Missouri, US) or Synthego (California, US).

#### Peptide prediction tools

Hydrophobic moments of peptides were calculated using the following peptide prediction software: http://rzlab.ucr.edu/scripts/wheel/wheel.cgi created by Don Armstrong and Raphael Zidovetzki. (Version: Id: wheel.pl,v 1.4 2009-10-20 21:23:36 don Exp). Tridimensional representations of PTD4 and CM18 independent peptides were obtained from the following peptide prediction software: http://zhanglab.ccmb.med.umich.edu/QUARK/. Tridimensional representations of CM18-PTD4 and 6His-CM18-PTD4 peptides were obtained from the following peptide prediction software: http://mobyle.rpbs.univ-paris-diderot.fr/cgi-bin/portal.py#forms::PEP-FOLD. Secondary structure predictions in S6 Table were performed with PSSpred (http://zhanglab.ccmb.med.umich.edu/PSSpred/) and Psipred (http://bioinf.cs.ucl.ac.uk/psipred/) softwares.

#### Cell nuclei counting tool

Nuclei with protein fluorescent signal in cells fixed for immunolabelling analysis were counted with the free software ImageJ: https://imagej.nih.gov/ij/.

#### Endosome escape assays

Microscopy-based and flow cytometry-based fluorescence assays were developed to study endosome leakage and to determine whether the addition of the shuttle agents facilitates endosome leakage of the polypeptide cargo. One day before the calcein assay was performed, cells in exponential growth phase were harvested and plated in a 24-well plate (80,000 cells per well). The cells were allowed to attach by incubating overnight in appropriate growth media, as described in **Example 1**. The next day, the media was removed and replaced with 300 μL of fresh media without FBS containing 2.5 mg/ml of FITC-Dextran (Sigma FD10S-100MG—10 kDa) with a red lysotracker Red DND-99 (100 nM) (Life Technologies L7528) for 30 min. At the same time, 6His-CM18-PTD4 peptide was added at a predetermined concentration. The plate was incubated at 37°C for 30 min. The cells were washed with PBS 1X (37°C) and fresh media containing FBS was added. The plate was incubated at 37°C for 2.5 hours. The cells were washed three times and were visualized by phase contrast and fluorescence microscopy (IX81^™^, Olympus).

#### Real-time Polymerase Chain Reaction (rt-PCR)

Control and treated cells are transferred to separate sterile 1.5-mL tubes and centrifuged for 5 min at 300 g. The cell pellets are resuspended in appropriate buffer to lyse the cells. RNAase-free 70% ethanol is then added followed by mixing by pipetting. The lysates are transferred to an RNeasyTM Mini spin column and centrifuged 30 seconds at 13000 RPM. After several washes with appropriate buffers and centrifugation steps, the eluates are collected in sterile 1.5-mL tubes on ice, and the RNA quantity in each tube is then quantified with a spectrophotometer. For DNase treatment, 2 μg of RNA is diluted in 15 μL of RNase-free water. 1.75 μL of 10X DNase buffer and 0.75 μL of DNase is then added, followed by incubation at 37°C for 15 min. For reverse transcriptase treatment, 0.88 μL of EDTA (50 nM) is added, followed by incubation at 75°C for 5 min. In a PCR tube, 0.5 μg of DNase-treated RNA is mixed with 4 μL of iScriptTM Reverse transcription Supermix (5X) and 20 μL of nuclease-free water. The mix is incubated in a PCR machine with the following program: 5 min at 25°C, 30 min at 42°C and 5 min at 85°C. Newly synthesized cDNA is transferred in sterile 1.5-mL tubes and diluted in 2 μL of nuclease-free water. 18 μL per well of a qPCR machine (CFX-96TM) mix is then added in a PCR plate for analysis.

#### Flow cytometry analysis

GFP signal intensity and GFP positive cell counting was quantified using flow cytometry (Accuri C6, Becton, Dickinson and Company (BD)). Untreated cells were used to establish a baseline in order to quantify the increased fluorescence due to the internalization of the fluorescent protein in treated cells. The percentage of cells with a fluorescence signal above the maximum fluorescence of untreated cells, “mean %” or “Pos cells (%)”, is used to identify positive fluorescent cells. “Relative fluorescence intensity (FL1-A)” corresponds to the mean of all fluorescence intensities from each cell with a fluorescent signal after fluorescent protein delivery with the shuttle agent. Also, the events scanned by flow cytometry corresponding to cells (size and granularity) were analyzed. The cellular toxicity (% cell viability) was monitored comparing the size (FSC) and the coarseness (SSC) of each cell delivery condition to untreated cells. Cell delivery conditions included the “cargo alone” control.

#### DNA signal intensities quantification

We used a Bio-Rad ImageLabTM software (Version 5.2.1, Bio-Rad, http://www.bio-rad.com/en-ca/product/image-lab-software?tab=Download) to quantify the relative signal intensities of each of the different bands directly on gels. The sum of the three bands of interest (one gene target and two cleavage products) in a given lane corresponds to 100% of the signal, and the numerical value at the bottom of each lane is the sum of the relative signals (%) of only the two cleavage product bands (black arrows). No cleavage product bands were found in the negative controls (“- ctrl”, i.e., to cells that were exposed to CRISPR/Cas9-NLS complex in the absence of shuttle agent).

#### Fluorescence microscopy analysis

The delivery of fluorescent protein cargo in cytosolic and nuclear cell compartments was observed with an Olympus IX70TM microscope (Japan) equipped with a fluorescence lamp (Model U-LH100HGAPO) and different filters. The Olympus filter U-MF2TM (C54942-Exc495/Em510) was used to observe GFP and FITC-labeled antibody fluorescent signals. The Olympus filter HQ-TRTM (V-N41004-Exc555-60/Em645-75) was used to observe mCherryTM and GFP antibody fluorescent signals. The Olympus filter U-MWU2TM (Exc330/Em385) was used to observe DAPI or Blue Hoechst fluorescent signals. The cells incubated in 50 μL of fresh medium were directly observed by microscopy (Bright-field and fluorescence) at different power fields (4x to 40x). The cells were observed using a CoolSNAP-PROTM camera (Series A02D874021) and images were acquired using the Image-ProplusTM software.

#### Cell immune-labelling

Adherent cells were plated on a sterile glass strip at 1.5x105 cells per well in a 24-plate well and incubated overnight at 37°C. For fixation, cells were incubated in 500 μL per well of formaldehyde (3.7% v/v) for 15 min at room temperature, and washed 3 times for 5 min with PBS 1X. For permeabilization, cells were incubated in 500 μL per well of Triton^™^ X-100 (0.2%) for 10 min at room temperature, and washed 3 times for 5 min with PBS. For blocking, cells were incubated in 500 μL per well of PBS 1X containing 1% BSA (PBS/BSA) for 60 min at room temperature. Primary mouse monoclonal antibody was diluted PBS/BSA (1%). Cells were incubated in 30 μL of primary antibody overnight at 4°C. Cells were washed 3 times for 5 min with PBS. Secondary antibody was diluted in PBS/BSA (1%) and cells were incubated in 250 μL of secondary antibody 30 min at room temperature in the dark. Cells were washed 3 times for 5 min with PBS and fixed with paraformaldehyde (4%). Glass strips containing the cells were mounted on microscope glass slides with 10 μL of the mounting medium Fluoroshield^™^ with DAPI. HoxB4 was labelled with a primary mouse anti-HOXB4 monoclonal antibody (Novus Bio #NBP2-37257) diluted 1/500. Cas9-NLS was labelled with a primary mouse anti-Cas9 monoclonal antibody (Millipore #MAC133) diluted 1/500. Cpf1-NLS was labelled with a primary anti-Cpf1 polyclonal antibody (Agrisera #AS16 3841) diluted 1/500. The same secondary anti-mouse antibody Alexa^™^-594 (Abcam #150116) diluted 1/1000 was used.

#### Western blot assay

Western blot detection of 6His-CM18-PTD4 required the adding of a His-Tag in C-terminal position. After 1 min incubation of the peptide at 10 μM with HeLa cells, cells were washed three times with PBS1X before lysis with RIPA lysis buffer (750 mM NaCl, 5% IGEPAL CA-630, 2.5% sodium deoxycholate, 0.5% SDS and 250 mM Tris pH 7.5). Cells were starved in eppendorf for centrifugation (13000 rpm at 4°C for 5 min). Lysates were assayed and incubated with Ni-NTA beads at 4°C over night. Beads were centrifuged (800g for 2 min), washed five times with RIPA buffer and resuspended in 2X SDS-PAGE buffer at 100°C for 10 min. Samples were centrifuged (800 g for 2 min) before loading on 16% tricine gel.

#### Resazurin assay

The viability of cells was assessed with a resazurin test. Resazurin is a sodium salt colorant that is hydrolyzed by mitochondrial enzymes in metabolically active cells. Resulting colorimetric conversion, which only occurs in viable cells, can be measured by spectroscopy analysis in order to quantify the percentage of viable cells. The stock solution of resazurin was prepared in water at 1 mg/100 mL and stored at 4°C. 25 μL of the stock solution was added to each well of a 96-well plate, and cells were incubated at 37°C for one hour before spectrometry analysis. The incubation time used for the resazurin enzymatic reaction depended on the quantity of cells and the volume of medium used in the wells.

#### Sytox red dead cell stain

Cell death was estimated with SYTOX^®^ Red dead cell stain is a high-affinity nucleic acid stain that easily penetrates cells with compromised plasma membranes but will not cross uncompromised cell membranes. Sytox was used at 5 nM and incubated on cells for 15 min at room temperature protected from light. Sytox Red labelling was observed by fluorescent microscopy and was measured by flow cytometry.

#### FITC-Annexin V assay for apoptosis detection

The human vascular anticoagulant, annexin V, is a 35–36 kD Ca2+-dependent phospholipid-binding protein that has a high affinity for PS.9 Annexin V labeled with a fluorophore or biotin can identify apoptotic cells by binding to PS exposed on the outer leaflet. Cells were washed with PBS 1X and incubated with 5 μL of FITC-Annexin-V in 100 μL in annexin-binding buffer for 15 min at room temperature. 400 μL of annexin-binding buffer was added and samples were kept on ice before flow cytometry analysis.

#### Endocytosis inhibitor assay

Each endocytosis inhibitor was incubated with cells for 1 h before protein delivery and kept in the medium during the process.

#### T7E1 assay

The T7 endonuclease I (T7E1) can be used to detect on-target CRISPR/Cas genome editing events in cultured cells. As an overview, genomic DNA from target cells is amplified by PCR. The PCR products are then denatured and re-annealed to allow heteroduplex formation between wild-type DNA and CRISPR/Cas-mutated DNA. T7E1, which recognizes and cleaves mismatched DNA, is used to digest the heteroduplexes. The resulting cleaved and full-length PCR products are visualized by gel electrophoresis. The T7E1 assay was performed with the Edit-R^™^ Synthetic crRNA Positive Controls (Dharmacon #U-007000–05 and IDT #1075932) and the T7 Endonuclease I (NEB, Cat #M0302S). After the delivery of the CRISPR/Cas complex, cells were lysed in 100 μL of Phusion^™^ High-Fidelity DNA polymerase (NEB #M0530S) laboratory with additives. The cells were incubated for 15–30 min at 56°C, followed by deactivation for 5 min at 96°C. The plate was briefly centrifuged to collect the liquid at bottom of the wells. 50-μL PCR samples were set up for each sample to be analyzed. The PCR samples were heated to 95°C for 10 min and then slowly (>15 min) cooled to room temperature. PCR product (~5 μL) was then separated on an agarose gel (2%) to confirm amplification. 15 μL of each reaction was incubated with T7E1 nuclease for 25 min at 37 °C. Immediately, the entire reaction volume was run with the appropriate gel loading buffer on an agarose gel (2%).

## Results and discussion

### 6His-CM18-PTD4 safely and efficiently delivers GFP-NLS to multiple mammalian cell types

We used the CPP HIV TAT-derived PTD4 and the ELD CM18 as templates to design ten PTD4/CM18-based peptide variants containing 0, 3, 6, 9 or 12 histidine residues in N- and/or C-terminal positions, or inside sequences (S1 Table). A recombinant green fluorescent protein (GFP) fused to a nuclear localization signal (NLS) was used as a functional fluorescent reporter system to measure a proper nuclear GFP signal distinct from endosomal entrapment [22]. Transduction efficiency of each CM18/PTD4-based peptide was tested by delivering GFP-NLS to HeLa cells (.

**Fig 1:**
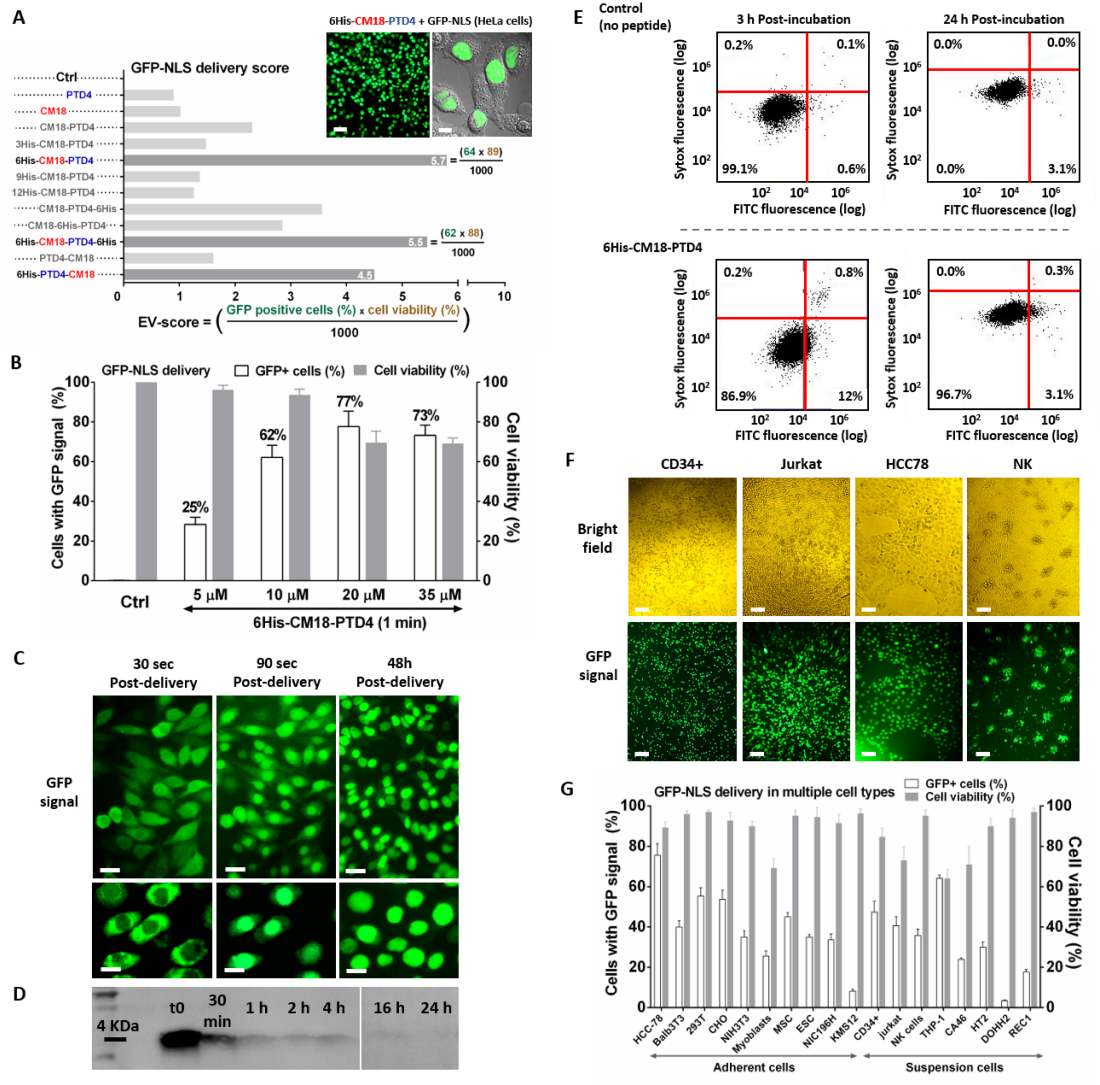
6His-CM18-PTD4 efficiently and safely delivers GFP-NLS into the cytosol after one-minute co-incubation with various mammalian cells. (A) EV_Score for CM18/PTD4-based peptide variants (10 μM) that delivered GFP-NLS (10 μM) in HeLa cells after 1 min co-incubation. Fluorescence (left panel) and confocal (right panel) microscopy analysis of GFP-NLS delivery with 6His-CM18-PTD4 (10 μM) in HeLa cells (Scale bars: microscopy—50 μm; confocal—10 μm). (B) Flow cytometry analysis of HeLa cells incubated for 1 min with different concentrations of 6His-CM18-PTD4 (5 μM to 35 μM) and GFP-NLS (10 μM). (C) Real time imaging capture of HeLa cells 30 sec and 90 sec after co-incubation with GFP-NLS (10 μM) and 6His-CM18-PTD4 (10 μM) (Scale bars: upper panels—20 μm; bottom panels—10 μm). (D) One minute after an incubation of 6His-CM18-PTD4 (10 μM) with HeLa cells, a His-Tag immuno-labelling was performed on cells and peptide degradation was observed over 24 h. (E) Cell viability assay with both the pre-apoptosis reporter FITC-Annexin V and the Sytox red reagents were performed 3 or 24 hours after 1 min incubation of HeLa cells with 6His-CM18-PTD4 (10 μM). (F) Microscopy analysis of the GFP-NLS (10 μM) delivery by 6His-CM18-PTD4 (10 μM) in HCC78 cells or by 6His-CM18-PTD4 (5 μM) in human CD34+, Jurkat, and NK cells (Scale bars 50 μm). (G) GFP-NLS (10 μM) delivery efficiency and related cell viability in multiple mammalian cells after 1 min incubation of 6His-CM18-PTD4 (10 μM) with adherent cells or of 6His-CM18-PTD4 (5 μM) with cells in suspension.

To classify peptide efficiency, GFP-NLS fluorescence and cell viability were converted in a single numerical value, calculated from flow cytometry data, and named “Entry-Viability-Score or EV-Score” as described in Eq 1 (Fig 1A and Part A of S1 Fig).

$$EV\_Score=\frac{\left(entry\%*viability\%\right)}{1000}$$ (1)

Amongst PTD4/CM18-based peptide variants (S1 Table), the EV-Score revealed that linking a domain with 6 consecutive histidines to the CM18-PTD4, compared to 0, 3, 9 or 12 residues, greatly improved the delivery without affecting viability. Following a 1 min co-incubation of GFP-NLS (10 μM) and the 6His-CM18-PTD4 (10 μM) with HeLa cells, 64% of GFP-positive cells with 89% viability were measured resulting in a EV-score of 5.7 (64*89/1000 = 5.696). Fluorescence microscopy and confocal imaging confirmed that GFP-NLS avoided endosomal entrapment and reached the nucleus of the HeLa cells. No change in cell morphology was observed, supporting the measured cell viability (Fig 1A—upper right panels and Part B of S1 Fig).

We tested concentrations of 5 to 35 μM of6His-CM18-PTD4 peptide, the best candidate for protein delivery, on HeLa cells. A peptide concentration of 10 μM provided the most efficient GFP-NLS delivery without significant toxicity (Fig 1B and Parts C and D of S1 Fig). To study delivery entry kinetics, real-time microscopy imaging was used. This showed that co-incubation of 6His-CM18-PTD4 and GFP-NLS in HeLa cells leads to GFP-NLS delivery to the cytosol after 30 sec followed by nuclear import 1 min later (Fig 1C).

Western blot analysis using an anti-6x histidine tag antibody showed that 6His-CM18-PTD4 was almost undetectable 1 h after exposure to HeLa cells. Under the same conditions, GFP-NLS remains in the nucleus for at least 48 h (Fig 1C and 1D). Three hours after GFP-NLS delivery with 10 μM 6His-CM18-PTD4, HeLa cell mortality was quantified with the cell-impermeable sytox red stain and the pre-apoptotic reporter fluorescein isothiocyanate (FITC)-Annexin-V. Flow cytometry analysis showed that only 0.8% of HeLa cells were permeable to sytox red and 12% were labeled by FITC-Annexin-V (Fig 1E). When we increased the concentration to 15 μM, 6His-CM18-PTD4 induced an increase of the pre-apoptosis Annexin V signal to 68% 3h after the delivery (Figure E in S1 Fig). Twenty-four hours after cell exposure to the peptide at 10 μM for 1 min, only 3.1% of HeLa cells had affinity for Annexin V, confirming that these conditions were optimal for protein delivery to HeLa cells. Afterward, GFP-NLS delivery was used for 18 mammalian cell types (Fig 1F and 1G, Part F of S1 Fig and S3 Table). We observed that cells cultivated in suspension were more brittle when exposed to the 6His-CM18-PTD4. Consequently, the peptide concentration was reduced to 5 μM with a 30 sec incubation time.

Depending on the cell types, we observed that 6His-CM18-PTD4 promotes rates of GFP-NLS delivery efficiency from 15% to 75% among 18 cell types. 6His-CM18-PTD4 had a low delivery efficiency in only two cell types, DOHH2 and KMS12 cells, in which the GFP signal was 3.5% and 8.1%, respectively (Fig 1G and Part F of S1 Fig).

### 6His-CM18-PTD4 enables repeated HoxB4 transcription factor delivery to maintain gene regulation

Following the successful delivery of GFP-NLS to multiple cell types, we investigated if the 6His-CM18-PTD4 peptide could efficiently deliver functional proteins with gene-regulating activity. The delivery of recombinant transcription factors in therapeutic cells is a promising method to transiently regulate gene expression [23]. The transcription factor Homeobox (Hox) HoxB4 is a potential candidate for hematopoietic stem cell expansion (S4 Table) [24, 25]. The direct delivery of the HoxB4 recombinant protein thus seemed to be a promising strategy to promote the proliferation of hematopoietic stem cells (HSCs) [25]. However, because of the short half-life of HoxB4 (approximately one hour), protocols were developed for repeated rounds of HoxB4 delivery in order to maintain long-lasting gene regulation. In that way, HoxB4 was engineered as degradation-resistant to improve its half-life in cells [25] but its adaptation to conditions meeting the standards used in cell therapy units was not validated due to the unreliability of delivery methods (e.g., retroviral vectors). Furthermore, HoxB4 recombinant protein was fused to the CPP Human Immunodeficiency Virus (HIV)-TAT to promote its intracellular uptake [26]. Although the TAT-HoxB4 strategy was validated as a proof of concept resulting in HSC expansion, using a CPP-protein complex still poses the problem of endosomal entrapment after intracellular uptake by endocytosis [25, 27, 28].

**Fig 2:**
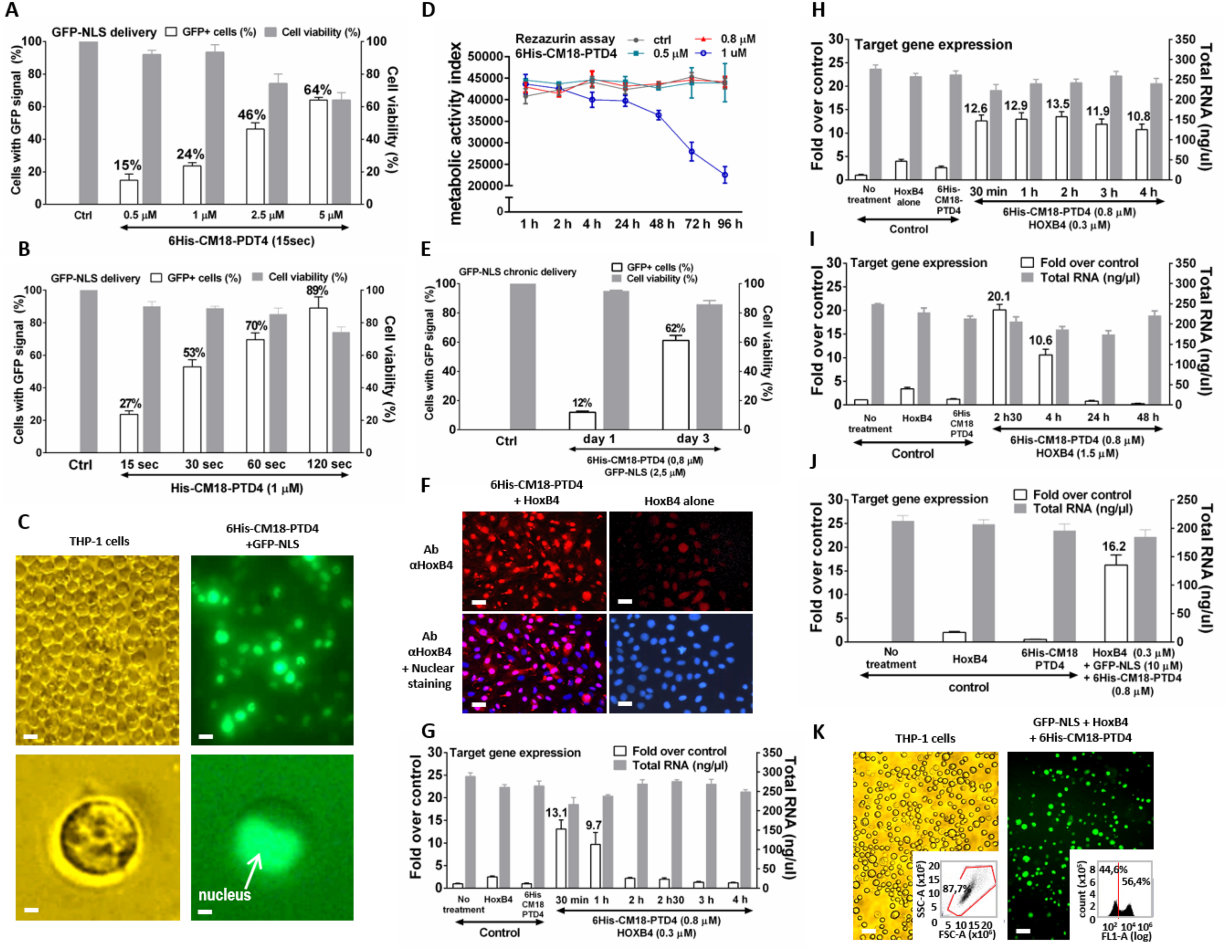
Successful repeated deliveries of the HoxB4 transcription factor with 6His-CM18-PTD4 maintain gene regulation in human monocytes THP1. (A) Flow cytometry analysis of THP-1 cells incubated for 15 sec with different concentrations of 6His-CM18-PTD4 (0.5 μM to 5 μM) and GFP-NLS (10 μM). (B) Flow cytometry analysis of THP-1 cells incubated for 15 sec to 120 sec with 6His-CM18-PTD4 (1 μM) and GFP-NLS (10 μM). (C) Fluorescence microscopy analysis of THP-1 cells incubated with 6His-CM18-PTD4 (1 μM) and GFP-NLS (10 μM) for 30 sec (Scale bars: upper panels—20 μm; bottom panels—2 μm). (D) Quantification of rezazurin hydrolysis 1 hour to 96 hours after incubation of different concentrations of 6His-CM18-PTD4 (0.5 μM to 1 μM) with THP-1 cells. (E) Flow cytometry analysis of THP-1 cells continuously incubated for 1 day or 3 days with 6His-CM18-PTD4 (0.5 μM) and GFP-NLS (2.5 μM). (F) HeLa cells were fixed and permeabilized prior to immune-labelling of HoxB4 with specific antibody (red) and nuclear staining (Hoestch) after one-minute co-incubation with 6His-CM18-PTD4 (10 μM) and HoxB4 (7 μM) (Scale bars: 100 μm). (G) Real-time PCR analysis of the EGR1 gene expression performed 30 min to 4 h after a 30 min co-incubation of 6His-CM18-PTD4 (0.8 μM) and HoxB4 (0.3 μM) with THP1 cells in medium with serum. (H) Real-time PCR analysis of the EGR1 gene expression performed 30min to 4 hours after a 30 min co-incubation of 6His-CM18-PTD4 (0.8 μM) and HoxB4 (0.3 μM) with THP1 cells in medium with serum. A second 6His-CM18-PTD4-mediated HoxB4 delivery with the same experimental conditions was performed in the same cells 30 min to 4 hours after the first one and real-time PCR analysis was processed 30 min after the second HoxB4 delivery. (I) Real-time PCR analysis of the EGR1 gene expression performed 2.5 hrs, 4 hrs, 24 hrs and 48 hrs after a 30 min co-incubation of 6His-CM18-PTD4 (0.8 μM) and HoxB4 (1.5 μM) with THP1 cells in medium with serum. (J) Real-time PCR analysis of the EGR1 gene expression performed 30 min after a 30 min co-incubation of 6His-CM18-PTD4 (0.8 μM), GFP-NLS (10 μM) and HoxB4 (0.3 μM) with THP1 cells in medium with serum. (K) Fluorescence microscopy analysis of THP-1 cells incubated with GFP-NLS (10 μM), HoxB4 (0.3 μM) and 6His-CM18-PTD4 (0.8 μM) for 30 min (Scale bars: 10 μm). Flow cytometry raw data show the THP1 cell integrity depending size and coarseness (left panel) and the percentage of cells with a GFP positive signal (right panel).

Since HoxB4 has a short half-life of 1 h with fleeting activity [25], we adapted the method with deliveries at regular intervals using human monocyte THP1 blood cell model [29]. First, GFP-NLS delivery in THP1 was optimized for the function of peptide concentrations and incubation times (Fig 2A and 2B). Optimal efficiency was reached with 1 μM 6His-CM18-PTD4 and 1 min incubation time allowing a 70% GFP-NLS delivery with no observable toxicity (Fig 2A and 2C, and Parts A and C of S2 Fig).

Then, cell viability was measured following daily additions of 6His-CM18-PTD4s at three different concentrations (0.5 μM, 0.8 μM or 1 μM) in a medium with serum. The measurement was taken after 96 h with a standard resazurin assay. This assay showed that 6His-CM18-PTD4 at 0.8 μM does not impair the THP1 metabolic activity over a period of 96 h of incubation (Fig 2D). Finally, using 0.8 μM 6His-CM18-PTD4, GFP-NLS (2.5 μM) was delivered once daily over 72 h, to show an increasing of GFP-NLS fluorescence over time (Fig 2E). This confirms that it is possible to safely deliver a protein multiple times to the same cell population to sustain or increase its concentration.

To confirm that HoxB4 reaches the nucleus after 1 min delivery with 0.8 μM 6His-CM18-PTD4, an immunofluorescence assay in HeLa cells was performed. HoxB4 was observed in the nucleus, and it was observed that the signal decreased after 30 min (Fig 2F and Part F of S2 Fig) [26].

A post-delivery analysis was performed in THP1 cells to measure the duration of the transcriptional effect of HoxB4 (0.3 μM) by measuring EGR1’s mRNA level. Using 6His-CM18-PTD4 to deliver HoxB4, the EGR1 mRNA level was more than 5 times higher (13.1 fold) than using HoxB4 alone (2.5 fold) 30 min post-delivery (Fig 2G). HoxB4 possesses a homeodomain with translocation potential and can penetrate into cells by itself explaining the low level of gene activation observed in the control [30]. One hour post-delivery, gene transcription was reduced to 9.7 fold, and it stopped completely after two hours. An additional HoxB4 delivery performed 1 to 4 h after the first one restored gene transcription, demonstrating that it is possible to sustain HoxB4 transcriptional activity by multiple deliveries (Fig 2H).

Since the 6His-CM18-PTD4 and HoxB4 concentrations can be independently modulated (Figure G in S2 Fig), the concentration of HoxB4 was increased to 1.5 μM and gene expression was extended for more than 4 h with one single delivery (Fig 2I). Finally, since cell therapy processes may require the action of several transcription factors at the same time, simultaneous delivery of two proteins was investigated to THP1 cells. The co-delivery of GFP-NLS and HoxB4 with 6His-CM18-PTD4 resulted in GFP nuclear signal in 56.4% of cells and a HoxB4-mediated gene transcriptional modulation around 4 times higher than in the control condition (Fig 2J and 2K). Together, these results suggest that 6His-CM18-PTD4 peptide can be used to regulate gene transcription by delivering recombinant transcription factors. Because 6His-CM18-PTD4 was independent from the protein in our assays, it was possible to titrate the amount of protein delivered while maintaining a constant 6His-CM18-PTD4 concentration. In that way, optimal delivery activity of the peptide was sustained while transcriptional activity was independently modulated. The simultaneous co-delivery of HoxB4 and GFP-NLS further indicated that the 6His-CM18-PTD4 peptide could be used in applications requiring the delivery of more than one transcription factor [31].

### 6His-CM18-PTD4 delivers functional CRISPR Cas9/Cpf1 RNP complexes in hard-to-modify NK cells

While electroporation allows for high-efficiency delivery of Cas9 RNP to some therapeutic cells such as T-cells, other types of cells, namely NK cells, do not respond to this approach [4]. These hard-to-modify cells would highly benefit from new delivery methods. Consequently, we investigated the potential of 6His-CM18-PTD4 to deliver CRISPR-Cas9 and -Cpf1 RNPs to human T-cell-derived Jurkat and primary NK cells [32, 33].

**Fig 3:**
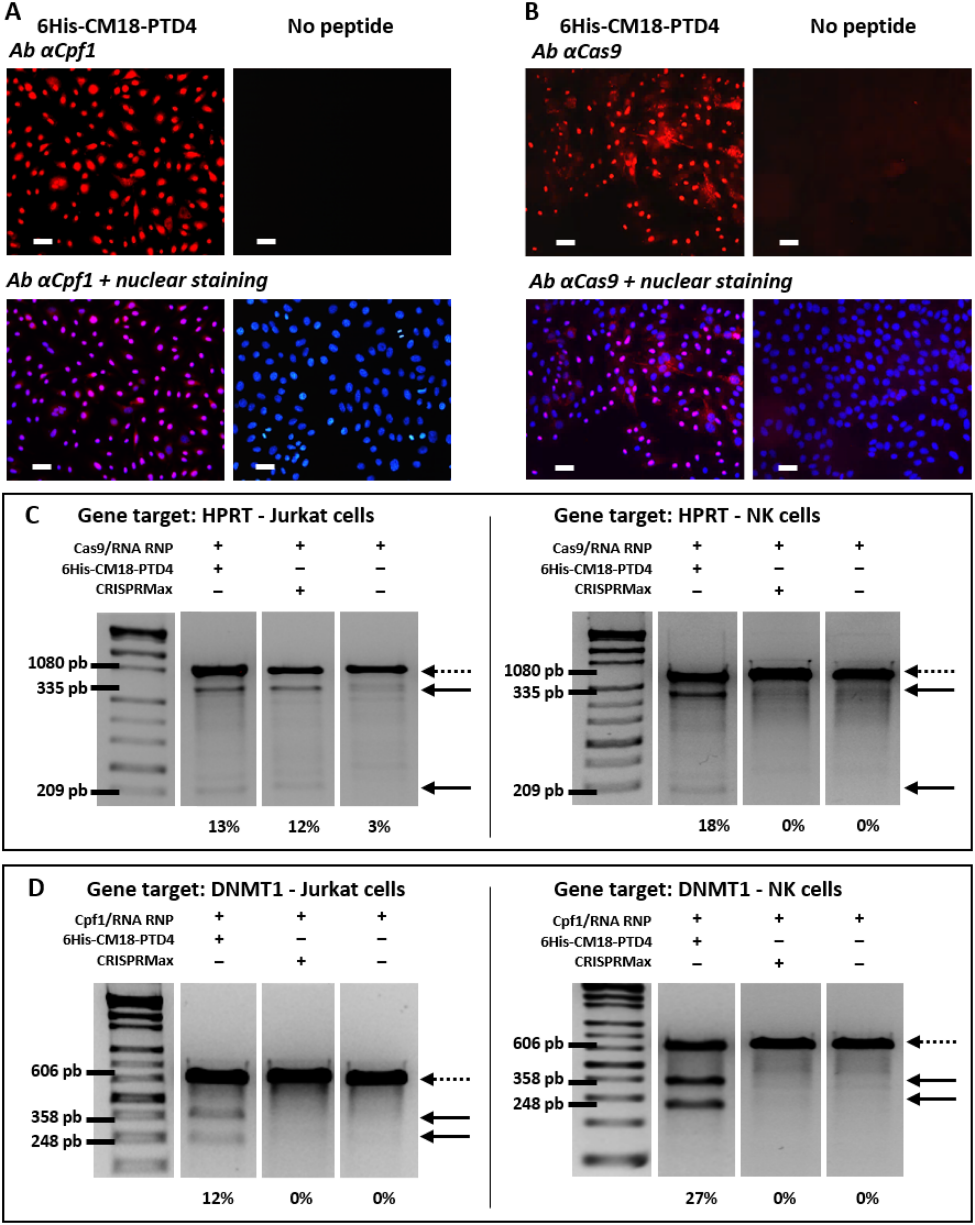
Robust genome editing after 6His-CM18-PTD4-mediated delivery of CRISPR/Cas9 and CRISPR/Cpf1 RNP complexes in HeLa, Jurkat and NK cells. (A-B) Fluorescence microscopy analysis of HeLa cells after a one-minute co-incubation with 6His-CM18-PTD4 (20 μM) and (A) Cas9-NLS RNP or (B) Cpf1-NLS RNP. HeLa cells were fixed and permeabilized prior to immuno-labelling with specific antibodies (red) targeting Cas9 and Cpf1, and cell nuclei were stained with Hoechst reagent. Negative control show HeLa cells after exposure to CRISPR RNPs without the presence of the peptide. Cas9-NLS was complexed with tracrRNA and crRNA targeting the HPRT gene. Cpf1-NLS was complexed with crRNA targeting the B2M gene (Scale bars: 50 μm). (C-D) PCR analysis on electrophoresis agarose gel of genomic cleavage products resulting from (C) HPRT gene editing with CRISPR/Cas9 RNP complex, and from (D) DNMT1 gene editing with CRISPR/Cpf1 RNP complex in Jurkat and NK cells. Jurkat or NK cells were incubated for 90 sec with 6His-CM18-PTD4 (4 μM) and CRISPR RNP complexes (Cas9-NLS 2.5 μM; tracrRNA 2 μM; crRNA 2 μM and Cpf1-NLS 1.33 μM; crRNA 2 μM) and harvested 48 hours post-delivery. Commercial lipid transfection reagent CRISPRMax was used as alternative delivery methods. Dotted arrows indicate the bands corresponding to the target gene, and thick black arrows indicate the bands corresponding to related cleavage products. For each gene target, the sum of the relative signal intensities of the two cleavage product bands was compared to the relative signal intensity of the wild-type gene (100%), and was indicated as an indel value (%) at the bottom of each panel.

First, we produced recombinant spCas9 and asCpf1 proteins in fusion with a single C-terminal nuclear localization signal to improve import into the nucleus (thereafter referred as Cas9-NLS and Cpf1-NLS). To validate the proper nuclear localization of these nucleases after delivery, 6His-CM18-PTD4 (20 μM) was co-incubated for 1 min with Cas9-NLS (2.5 μM) or Cpf1-NLS (1.33 μM), both complexed with their respective crRNAs. This experiment was performed in an adherent HeLa cell line to facilitate the visualization of the nucleus. An immunolabelling assay showed that Cas9-NLS and Cpf1-NLS successfully reached the nuclei of 60% and 96% of cells, respectively (Fig 3A and 3B, S3 Fig and S5 Table).

To measure the editing activity in Jurkat and NK cells, two crRNAs were synthetically produced to target either HPRT or DNMT1 genes with Cas9-NLS and Cpf1-NLS, respectively (see S4 Table for gene and guide sequences). For Cas9-NLS, the crRNA was used with a synthetically produced tracrRNA. Deliveries of both CRISPR RNPs were performed with either 6His-CM18-PTD4 or the commercial RNP delivery agent CRISPRMax. Genome editing results were analyzed using a T7 Endonuclease I assay. 6His-CM18-PTD4-mediated delivery of Cas9-NLS RNP induced 13% and 18% genome editing to Jurkat and NK cells, respectively (Fig 3C). The lipid agent CRISPRMax mediated the delivery of Cas9-NLS RNP to Jurkat cells, inducing 12% genome editing. When CRISPRMax mediated the delivery of Cas9-NLS RNP to NK cells, there was no genome editing. Interestingly, 6His-CM18-PTD4-mediated delivery of Cpf1-NLS RNP induced 12% and 27% in Jurkat and NK cells, respectively (Fig 3D). In contrast, no genome editing was observed after Cpf1-NLS RNP delivery with CRISPRMax to these cells.

Importantly, to judge the efficiency of this method for CRISPR RNP delivery, the crRNA we used to target the DNMT1 gene was the same sequence as the one encoded in a plasmid DNA vector tested in the easy-to-transfect HEK293 cells (Zetsche et al., 2015). One minute of co-incubation of Cpf1-NLS RNP and 6His-CM18-PTD4 with hard-to-modify NK cells resulted in similar robust DNMT1 gene editing level as in HEK293 and reached up to 27% (Fig 3D). As opposed to sustained expression of the CRISPR nuclease after intracellular DNA or even mRNA transfection [34], the single delivery of CRISPR RNP is an appealing method for cell therapy manufacturing [35–38]. Using a protein instead of DNA or mRNA reduces the exposure time of cells with the CRISPR nuclease and could dramatically reduce uncontrolled off-target effects, immune responses and plasmid vector integration into the genome [39–41]. Altogether, these results show that 6His-CM18-PTD4 is an efficient method to deliver CRISPR Cas9 and Cpf1 RNPs systems in several mammalian cell types and could enables the use of CRISPR recombinant proteins for therapeutic approaches.

### 6His-CM18-PTD4 co-delivers multiple CRISPR RNP systems in the same cells and promote directed homologous recombination

Using a DNA template with a CRISPR nuclease to precisely repair a genomic sequence is a promising approach to study and improve cell functions [42, 43]. We investigated the ability of 6His-CM18-PTD4 to deliver both the CRISPR RNP and a short single strand linear DNA template, simultaneously. The flexibility of 6His-CM18-PTD4 enabled the successful directed single-strand annealing repair of the HPRT and B2M genes after the delivery of single stranded DNAs co-incubated with the peptide and CRISPR systems in HeLa cells. Two strands were specifically designed with homology arms to insert in the HPRT or DNMT1 cleavage sites. HPRT-related DNA template sequence (72bp) was co-incubated with a Cas9-NLS RNP complex and 6His-CM18-PTD4.

**Fig 4:**
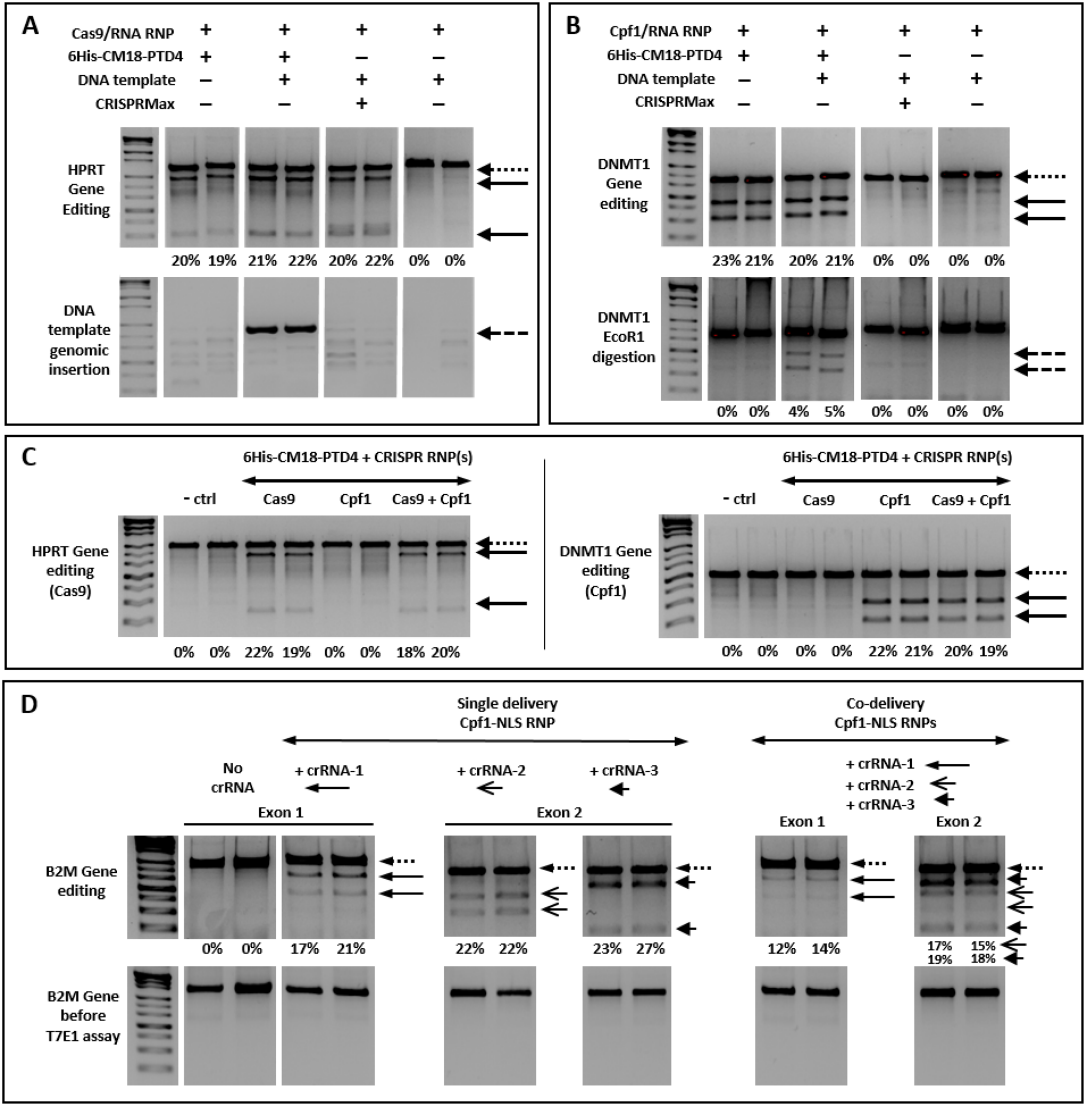
Successful directed recombination and multiple gene editing after co-incubating 6His-CM18-PTD4 and CRISPR RNPs with HeLa cells. (A-B) PCR analysis on electrophoresis gel of genomic cleavage products (thick arrows) of the (A) HPTR and (B) DNMT1 genes after the delivery of a specific CRISPR/Cas9-NLS or CRISPR/Cpf1-NLS RNP complexes respectively in HeLa cells. HeLa cells were incubated with 6His-CM18-PTD4 (35 μM), CRISPR RNP complexes (nuclease 0.5 μM; gRNA 0.4 μM) and DNA templates for 90 sec and harvested 48 hours post-delivery. Commercial lipid transfection reagent CRISPRMax was used as alternative delivery methods. Each short single strand DNA template (500 ng) was specifically designed with arm homology for its insertion in gene target cleavage site. 48 hours after CRISPR RNPs deliveries, (A) the genomic insertion of a DNA template (72 pb) in the Cas9-edited HPRT gene was confirmed by PCR amplification with specifically designed primers. (B) The genomic insertion of a DNA template (76 pb) containing an EcoR1 site in the Cpf1-edited DNMT1 gene was confirmed by exposing DNMT1 PCR product to a EcoR1 restriction enzyme (dashed arrow). (C) PCR analysis on electrophoresis gels of genomic cleavage product of the HPRT (left) and the DNMT1 (right) genes after the delivery of specific CRISPR/Cas9-NLS or CRISPR/Cpf1-NLS RNP complexes in HeLa cells. Hela cells were co-incubated for 90 sec with 6His-CM18-PTD4 (35 μM), a CRISPR/Cas9-NLS and/or a CRISPR/Cpf1-NLS RNP complexes (nuclease 1.25 μM; gRNA 1 μM) and harvested 48 hours post-delivery. (D) PCR analysis on electrophoresis gel of genomic cleavage products from three loci on the exons 1 and 2 of the B2M gene in HeLa cells. HeLa cells were incubated for 90 sec with 6His-CM18-PTD4 (35 μM) and the three CRISPR/Cpf1 RNP (crRNAs 1, 2 and 3) complexes together or separately, and harvested 48 hours post-delivery. Targeted genes before analysis are shown as negative control (bottom panels). Dotted arrows indicate the bands corresponding to the target gene, dashed arrows indicate band confirming the genomic insertion of DNA templates, and different thick black arrows indicate the bands corresponding to related cleavage products. For each gene target, the sum of the relative signal intensities of the two cleavage product bands was compared to the relative signal intensity of the wild-type gene (100%), and was indicated as an indel value (%) at the bottom of each panel.

The presence of the DNA template did not impair the delivery of Cas9-NLS RNP, nor its activity (Fig 4A), where an edition of 22% was observed in HeLa cells. Additionally, cleavage of the HPRT gene was followed by the successful integration of the DNA template at the genomic cleavage site, confirmed by DNA template amplification 48h after delivery. CRISPRMax-mediated delivery of Cas9-NLS RNP also resulted in HPRT gene editing but not in DNA template integration.

DNMT1 gene-directed editing was also performed in HeLa cells using a linear DNA template (76pb) containing an EcoR1 enzyme restriction site. Delivery of this template alongside with 6His-CM18-PTD4 and the Cpf1-NLS RNP resulted in the DNMT1 gene edition. The insertion of the DNA template was confirmed by the EcoR1 digestion of the DNMT1 gene amplified from HeLa cells 48h after delivery (Fig 4B). We observed no genome editing nor DNA template insertion for Cpf1-NLS RNP using the CRISPRMax protocol.

Next, the simultaneous delivery of 3 different CRISPR RNPs with 6His-CM18-PTD4 resulted in the editing of the 3 targeted genomic regions at once. we simultaneously co-delivered several CRISPR RNP complexes to HeLa cells to target different genome locations. 6His-CM18-PTD4 enabled the co-delivery of both Cas9-NLS and Cpf1-NLS RNPs that cleaved their respective HPRT and DNMT1 gene targets. Interestingly, genome editing levels achieved using RNP complexes together or individually resulted in similar efficiency (Fig 4C). Finally, the simultaneous co-delivery of three Cpf1-NLS RNP complexes targeting different loci of B2M gene was performed in HeLa cells. In this experiment, one crRNA (crRNA#1) targeted exons 1 and two others crRNAs (crRNA#2 and #3) targeted two loci into exon 2 of the B2M gene (Fig 4D and S4 Table). Simultaneous genome editing was successful indicating that 6His-CM18-PTD4 enables the efficient co-delivery of at least 3 different CRISPR RNPs.

### 6His-CM18-PTD4 activates translocation and endocytosis in a concentration-dependent manner

**Fig 5:**
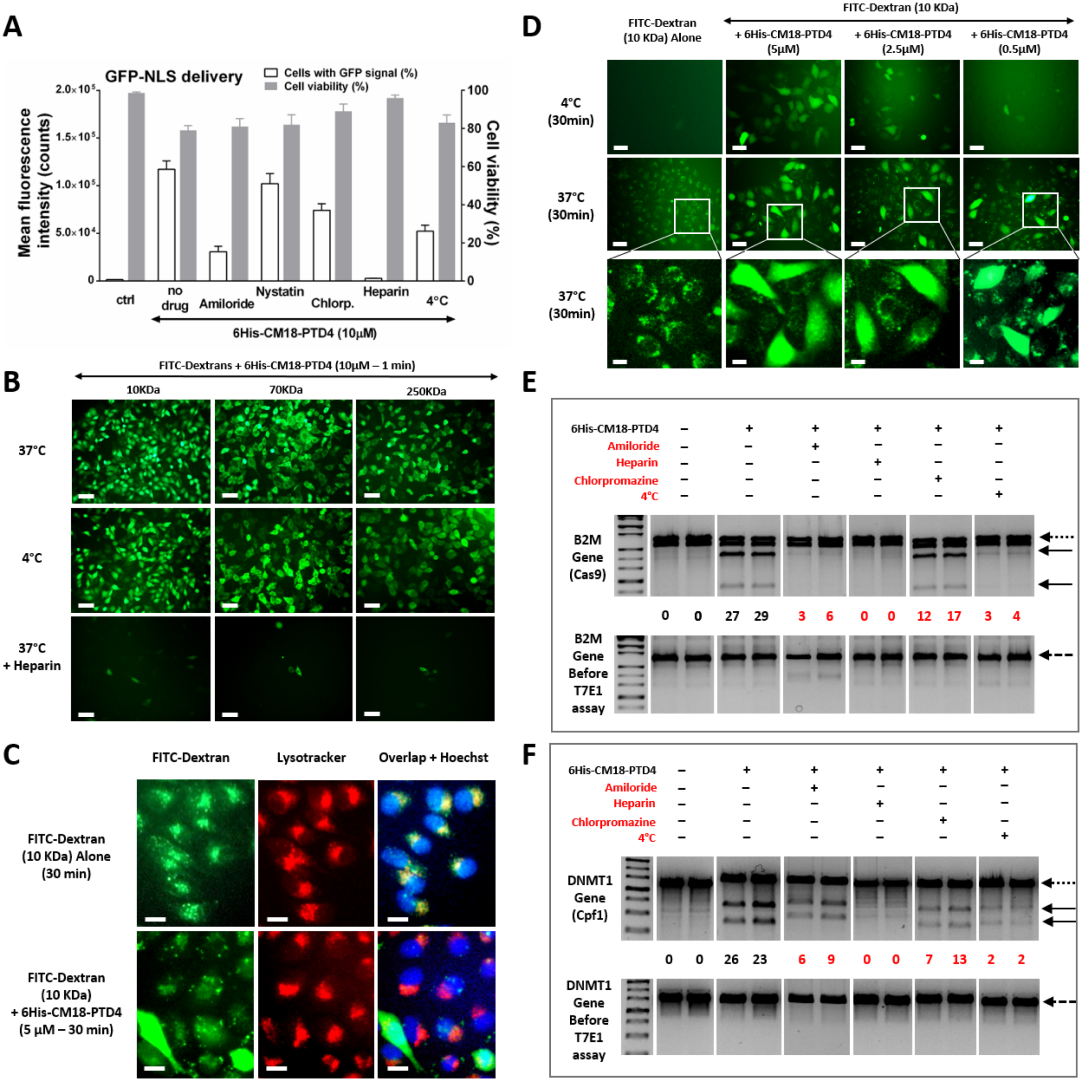
6His-CM18-PTD4 activates both translocation and endocytosis mechanisms. (A) Flow cytometry analysis of HeLa cells incubated for 1 min with GFP-NLS (10 μM) and 6His-CM18-PTD4 (10 μM) at 37°C or 4°C, and in presence of the macropinocytosis inhibitor amiloride, the caveolae-dependent endocytosis inhibitor nystatin, the clathrin-dependent endocytosis inhibitor chlorpromazine and the HSPG ligand heparin. (B) Fluorescence microscopy analysis of HeLa cells after a one-minute co-incubation with 6His-CM18-PTD4 (10 μM) and FITC-dextrans of different sizes (10, 70 and 250 KDa) at 37°C, 4°C or in presence of heparin (Scale bars: 100 μm). (C) Fluorescence microscopy analysis of HeLa cells after 30 min incubation with FITC-dextran (10 KDa) alone or after 30 min co-incubation with 6His-CM18-PTD4 (5 μM) and FITC-dextran (10 KDa). Lysosomes are labeled with a red lysotracker and nuclei are stained with Hoechst (Scale Scale bar: 15 μm). (D) Fluorescence microscopy analysis of HeLa cells after 30 min co-incubation at 4°C and 37°C with FITC-dextran (10 KDa) and different concentrations of 6His-CM18-PTD4 (5 μM, 2.5 μM and 0.5 μM) (Scale bars: upper and middle panels—20 μm; bottom panels—10 μm). (E-F) PCR analysis on electrophoresis gel of genomic cleavage product (thick arrows) from (E) B2M and (F) DNMT1 genes (dotted arrows) in HeLa cells. HeLa cells were co-incubated for 90 sec with 6His-CM18-PTD4 (20 μM) and (E) CRISPR/Cas9-NLS or (F) CRISPR/Cpf1-NLS RNP complex (nuclease 0.5 μM; gRNA 0.4 μM) and harvested 48 hours post-delivery. CRISPR RNPSs deliveries were performed at 37°C and 4°C, and in presence of amiloride, nystatin, chlorpromazine or heparin. Inhibitory conditions that affected genome editing are highlighted in red. Targeted genes before analysis are shown as negative control (bottom panels—dashed arrow).

To investigate the delivery mechanism and endosomolytic activity of 6His-CM18-PTD4, we selected endocytosis inhibitors that target specific pathways: amiloride is a macropinocytosis blocker, nystatin is a caveolae-dependent endocytosis blocker, and chlorpromazine is a clathrin-mediated endocytosis blocker. Furthermore, all forms of endocytosis were inhibited by exposing HeLa cells to low temperature (4°C). The same low temperature did not affect direct translocation through the cell membrane. We also used heparin, a close structural homologue of heparan sulfate proteoglycans (HSPGs), to compete with 6His-CM18-PTD4 for cell penetration mediated by HSPG at the cell surface. We investigated the impact of endocytosis inhibitors, heparin and low temperature on the delivery of the protein GFP-NLS. Flow cytometry analysis showed that the presence of nystatin did not block GFP-NLS delivery efficiently. In contrast, the presence of chlorpromazine or amiloride resulted in the reduction of signal intensity by around 2 to 4 times, respectively. GFP-NLS delivery was completely blocked after the co-incubation of 6His-CM18-PTD4 with heparin, indicating a potential affinity of the peptide for HSPGs (Fig 5A and Part A of S4 Fig). When all forms of endocytosis were blocked at 4°C, the GFP-NLS signal decreased approximately 2.5 times compared to the “No drug” condition (Fig 5A and Part A of S4 Fig). This partial loss of protein signal at low temperature suggests that 6His-CM18-PTD4 activates both translocation and endocytosis mechanisms.

To confirm the activation of these uptake mechanisms by the peptide, we performed further investigations with fluorescein isothiocyanate (FITC)-dextrans of various sizes. 6His-CM18-PTD4 at 10 μM enabled the delivery of the 10, 70 and 250 kDa FITC-dextrans after 1 min incubation with HeLa cells at 37°C and 4°C (Fig 5B). This suggests that 6His-CM18-PTD4 activated translocation in a size-independent manner.

Furthermore, the presence of heparin at 37°C abolished peptide-mediated delivery of 10, 70 and 250 kDa FITC-dextrans. Since natural endocytosis of FITC-dextran is a process requiring more than 1 min incubation with cells, the endosomolytic activity of the peptide was investigated using an extended incubation time (30 min) and a reduced peptide concentration (5 μM) to sustain cell viability. Thirty minutes after the incubation of 10 kDa FITC-dextran alone with HeLa cells at 37°C, microscopy analysis showed fluorescent signal emanating from endosomes and lysosomes (Fig 5C) demonstrating endocytosis of the dextran. When co-incubated with 6His-CM18-PTD4 (5 μM), FITC-Dextran signal was both endosomal and cytosolic but did not co-localize with the lysotracker. This indicates that 6His-CM18-PTD4 enabled the endosomal escape of the FITC-Dextran before reaching lysosomes.

The same experiments performed at 4°C, incubating FITC-dextran alone, resulted in the absence of endosomal fluorescent signal (Fig 5D). At 4°C, co-incubating FITC-dextran with 5 μM 6His-CM18-PTD4 (Figure B in S4 Fig) results in the translocation of the FITC-dextran. The decrease of the peptide concentration to 2.5 μM at 4°C resulted in the reduction of FITC-dextran delivery and, ultimately, in the lack of translocation using 0.5 μM 6His-CM18-PTD4. Interestingly, microscope analysis showed that, when FITC-dextran is endocytosed at 37°C, 6His-CM18-PTD4 enabled endosomal membrane destabilization and leakage of the endocytosed FITC-dextran even at 0.5 μM (Fig 5D, and Parts B and C of S4 Fig). Taken together, these results indicate that the activation of uptake pathways by 6His-CM18-PTD4 is concentration-dependent.

**Fig 6:**
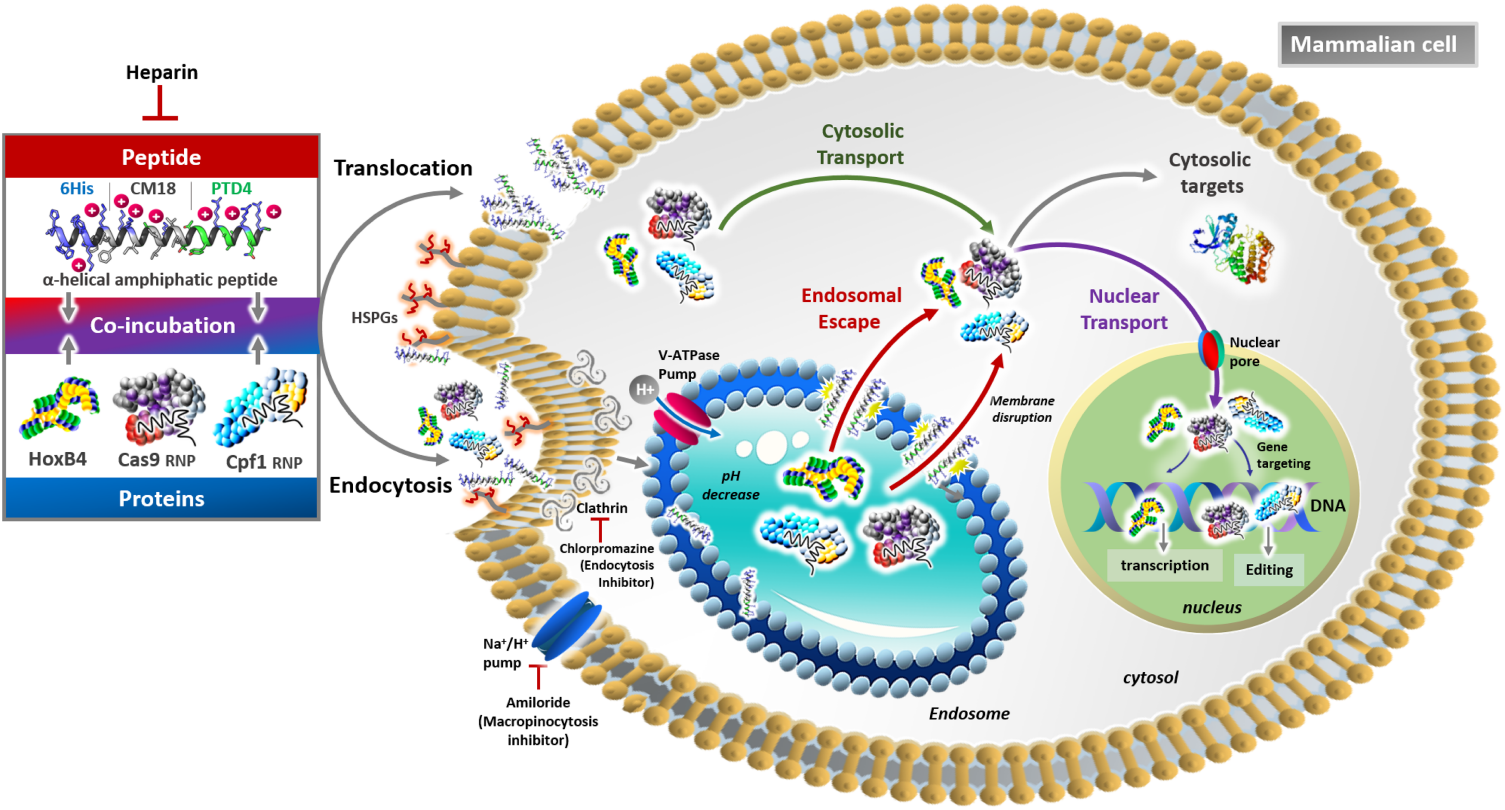
6His-CM18-PTD4 efficiently deliver proteins into the cytosol of mammalian cells. The 6His-CM18-PTD4 cationic helical amphiphilic peptide and proteins cargoes are mixed in PBS or culture medium with or without serum and are co-incubated for less than two minutes with mammalian cells. Multiple cell entrance mechanisms are activated and the nature of the cargo influences which path is followed to reach the cytosol. Activation of translocation leads to direct membrane permeation / poration resulting in the cytosolic delivery of proteins cargoes. 6His-CM18-PTD4 may also activate energy-dependent uptake like endocytic processes followed by the release of proteins from endosomes to the cytosol. The membrane lytic properties of the peptide destabilize endosomal membrane integrity. Histidine residues mediate the “proton sponge effect” in a pH dependent-manner and destabilize the endosomal membrane. Once in the cytosol, proteins are fully functional to reach their target and mediated biological outcomes. By concerns of clarity, molecules were not drawn at real scale.

Previous investigations have shown that the CM18 domain mediates membrane poration only at concentrations up to 3 μM to initiate translocation, and that the PTD4 domain specifically activates clathrin-dependent endocytosis [13, 44, 45]. Our results could explain why the combination of the 6 histidine, CM18 and PTD4 domains induces the co-activation of translocation and energy-dependent uptake mechanisms. Furthermore, six consecutive histidine residues has been shown to improve protein uptake and to bind HSPGs [46–48]. Our results also corroborate the compelling role of histidine and proteoglycans in the activation of macropinocytosis by 6His-CM18-PTD4 (Duchardt et al, 2007; Dixon et al, 2016). Indeed, whilst CM18 and PTD4 domains used separately are inefficient for protein delivery, 6His-CM18-PTD4 would have the functional synergy required for membrane destabilization (Fig 6).

In addition to concentration, the nature of the cargo may also influence the entry mechanism. Proteins and cells have heterogeneous properties that can influence the CPP-mediated delivery of macromolecule cargoes [49–51]. We therefore tested delivering Cas9 and Cpf1 RPNs in HeLa cells with 6His-CM18-PTD4 at 37°C and 4°C, or in presence of amiloride, chlorpromazine or heparin. Cleavage products of the B2M and DNMT1 targeted genes were approximately half-reduced with chlorpromazine, almost abolished with either amiloride or at 4°C, and no cleavage was observed with heparin (Fig 5E and 5F). These results suggest that the peptide mainly internalized CRISPR RNP complexes by macropinocytosis and clathrin-dependent endocytosis, and that translocation was not a prerequisite to deliver this type of protein cargo into the cytosol.

Physico-chemical factors like the size, the net charge and the isoelectric point of proteins may influence molecular interactions. Both endocytosis and direct translocation were involved in GFP-NLS (35 kDa) uptake while energy-dependent macropinocytosis and clathrin-mediated endocytosis were the predominant pathways for Cas9-NLS (170 kDa) and Cpf1-NLS (156 kDa) delivery. This suggests that protein size could influence uptake pathways activated by 6His-CM18-PTD4 [51]. However, 6His-CM18-PTD4 delivered 10, 70 and 250 kDa FITC-dextrans with similar efficiency in one minute, indicating that protein size was not the main property influencing uptake.

CRISPR RNPs convey negatively charged guide RNAs that may differently influence peptide membrane interactions compared to cargos with a positive net charge at physiological pH like GFP and HoxB4. Interactions between 6His-CM18-PTD4 and crRNA should be inhibitory and could explain why an increased concentration of 6His-CM18-PTD4 was required to deliver CRISPR RNPS compared to others cargoes. Similar inhibitory effects between arginine-rich cell-penetrating peptides and negatively charged molecules have been reported [28, 52, 53].

Finally, the isoelectric point (pI) of HoxB4 (9.8) is not suggestive of favorable electrostatic interactions with 6His-CM18-PTD4 (11.76). In contrast, GFP-NLS, which has a lower pI (6.3), could interact with the peptide at physiological pH. Since 6His-CM18-PTD4 efficiently delivers HoxB4 and GFP-NLS, it suggests that peptide-protein interaction was not a prerequisite for membrane permeation and the transport of the cargo to the inside of cells [28]. With this diversity of protein and membrane interactions, it remains remarkable that such different cargoes were successfully delivered by 6His-CM18-PTD4 and their role in uptake processes will require further investigation [15, 54, 55].

### Amphipathic level of 6His-CM18-PTD4 analogues correlate with protein delivery efficiency

Peptides with high amphiphilic levels are known to promote membrane permeabilization (Marie et al, 2014). The level of amphiphilicity of a peptide depends of the asymmetric repartition of hydrophilic and hydrophobic residues on its opposite faces, and is measured as the hydrophobic moment. For membrane perturbation, charge density and hydrophobic interactions between peptides and the membrane are critical [49, 56].

Regarding the diversity of cell types in which 6His-CM18-PTD4 efficiently delivered proteins, peptide membrane interactions are likely governed by non-specific electrostatic and hydrophobic interactions with ubiquitously expressed molecules, most likely negatively charged HSPGs, rather than specific receptor [57]. Whereas PTD4 and CM18 peptides taken separately have low hydrophobic moment (PTD4 2.44μ_H_, CM18 4.28 μ_H_) and low protein delivery efficiency, the fusion of CM18 with PTD4 resulted in peptides with heightened amphiphilic repartition of hydrophobic and hydrophilic residues along the helical axis (CM18-PTD4 6.72 μ_H_) (Figure A in S5 Fig and S6 Table).

Interestingly, changing the position of the PTD4 and CM18 domains did not affect the amphipathic level of the peptide, and analogues CM18-PTD4 and PTD4-CM18 shared similar protein delivery efficiency. In fact, we observed that the hydrophobic moment of each peptide analogue, including those with six histidine residues in N- and/or C-terminal position or therebetween, was correlated to their delivery activity (Figure B in S5 Fig) [58]. It indicates the important role of amphiphilicity in the protein delivery process orchestrated by the PTD4 and CM18 combination.

Adding a domain with six consecutive histidines residues to CM18-PTD4 and PTD4-CM18 peptides increased their hydrophobic moment and protein delivery efficiency (6His-CM18-PTD4 7.79 μ_H_). In addition to its buffering activity related to a “proton sponge effect” and its affinity for HSPGs [20, 55], histidine also has a structural role in peptide sequences.

Indeed, changing the position of the histidine tail in the peptide sequence induced different degrees of amphiphilicity correlated to changes in protein delivery efficiencies. The different positions of the histidine-rich domain inside peptide analogues may change the accessibility of histidine residues to membrane, which suggests that histidine membrane interaction regulate, at least in part, the activation of cell entry mechanisms. Indeed, each peptide analogue should be considered as a unique entity with its own delivery profile, and not only as the sum of two independent domains with distinct functions.

### 6His-CM18-PTD4 owns relevant features for cell therapy manufacturing

Here we showed that using a simple one-minute co-incubation protocol, the chemical-free and water-soluble peptide 6His-CM18-PTD4 efficiently mediates the intracellular delivery of various proteins with low toxicity. The absence of coupling between 6His-CM18-PTD4 and the protein allowed the proteins to keep their native conformation and full activity. This was also useful to modulate both cargo and 6His-CM18-PTD4 concentrations independently for optimal activity into cells, which is impossible when the peptide and protein are bound together. Both endosomolytic activity and, unexpectedly, direct translocation are mediated by 6His-CM18-PTD4 to bypass endosomal entrapment. This approach was successful in 20 mammalian cell types including stem cells, human primary cells, and cancer cell models, indicating the ability of 6His-CM18-PTD4 to activate universal uptake mechanisms with different transduction frequencies and kinetics between cell types.

The fast uptake activity of the soluble and chemical-free 6His-CM18-PTD4 peptide enables an almost instantaneous protein delivery and timing control to rapidly switch membrane-perturbing effects on and off in multiple cell types. Importantly, the peptide is efficient enough to deliver functional proteins at nano- and micromolar concentrations by co-incubation in medium with serum. Using independent peptides and proteins makes it possible to titrate the protein concentration independently of 6His-CM18-PTD4 and to modulate the amount of protein delivered to the cytosol and the biological output.

A further strength of the method is the large scope of cell types and materials that can be addressed and that, in other methods, usually require cargo covalent attachment to chemical or lipid reagents (Pisal et al, 2010). Structural properties, like helical conformation and amphiphilicity, confer unique peptide signatures that could provide useful guidelines for the rational design of amino acid sequences with protein delivery functions in mammalian cells. The 6His-CM18-PTD4 peptide has ideal features for next-generation intracellular delivery systems and highlight a trend towards direct protein delivery rather than protein expression from DNA/RNA vectors [2].

In conclusion, this peptide causes minimal cell perturbation, contains no harmful and hard-to-degrade chemical modifications, and exploits universal delivery mechanisms. Maintaining the native structure of the molecules to be delivered, compatibility with intracellular targeting, dosage control, and cost-effective designs are other main features required for high-throughput screening, clinical translation, good manufacturing practices, and large-scale manufacturing.

## Supporting information

S1 Table Peptide sequences listing and features. (DOCX)

S2 Table Recombinant protein sequences. (DOCX)

S3 Table Cell lines and culture conditions. (DOCX)

S4 Table Targeted genes and guide RNAs. (DOCX)

S5 Table Size of cleavage product fragments from Fig 3. (DOCX)

S6 Table Secondary structures prediction of PTD4, CM18, CM18-PTD4 and 6His-CM18-PTD4 peptides. (DOCX)

S1 Fig One-minute co-incubation with 6His-CM18-PTD4 results in efficient and safe delivery of GFP-NLS in multiple mammalian cells. (A) Flow cytometry raw data of HeLa cell viability depending size and coarseness (upper panels) and of GFP signal after GFP-NLS (10 μM) delivery (bottom panels). CM18 /PTD4 peptides analogues (10 μM) and GFP-NLS (10 μM) were co-incubated with HeLa cells for 1 min and flow cytometry analysis was performed 4 hours after GFP-NLS delivery. GFP positive cells are counted on the right of the red line threshold and cells without fluorescence are counted on the left. (B) Microscopy analysis of HeLa cells incubated with GFP-NLS alone. Microscopy images show the HeLa confluence and morphology in bright field (left panel) and the absence of GFP signal (right panel) (Scale bars: 50 μm). (C) Flow cytometry raw data of HeLa cell viability depending size and coarseness (upper panels) and of GFP signal after GFP-NLS (10 μM) delivery (bottom panels). Different concentrations of 6His-CM18-PTD4 (5 μM to 35 μM) and GFP-NLS (10 μM) were co-incubated with HeLa cells for 1 min and flow cytometry analysis was performed 4 hours after GFP-NLS delivery. (D) Flow cytometry analysis of HeLa cells co-incubated for 15 secs to 10 min with 6His-CM18-PTD4 (10 μM) and GFP-NLS (10 μM). Corresponding flow cytometry raw data are shown on the right of the panel. (E) Flow cytometry analysis of HeLa cells exposed to the pre-apoptosis marker FITC-Annexin V 3 h and 24 h after a one-minute incubation with different concentrations of 6His-CM18-PTD4 (2.5 μM to 15 μM). Fluorescence microscopy and flow cytometry analysis of multiple mammalian cell types (Balb3T3, 293T, CHO, NIH3T3, Myoblasts, MSC, ESC, NIC196H, KMS-12, THP1, CA46, HT2, DOHH2, REC-1 cells) incubated with 6His-CM18-PTD4 (10 μM for 1 min in adherent cells; 5 μM for 30 secs in suspension cells) and GFP-NLS (10 μM). Fluorescent microscopy analysis shows bright field views of cells (upper panels) and emanating GFP signal (bottom panels) (Scale bars: 100 μm). Flow cytometry analysis in the grey square relate to fluorescence microscopy images from CD34+, Jurkat, NK and HCC-78 cells in Fig 1G. (G) Fluorescence microscopy analysis of human primary myoblasts 24 hours after a one-minute co-incubation with 6His-CM18-PTD4 (10 μM) and GFP-NLS (10 μM). Cells were fixed and permeabilized prior to immuno-labelling with an anti-GFP antibody and a fluorescent secondary antibody to compensate the reduction of the GFP signal after 24 h. Immuno-labelled GFP is shown in left panel and overlapping with Hoechst staining show nuclei in right panel (Scale bars: 10 μm).
(TIF)

S2 Fig Fast and repeated GFP-NLS and HoxB4 delivery with 6His-CM18-PTD4 in THP1 cells. (A-B) Flow cytometry raw data of THP-1 cell viability depending size and coarseness (upper panels) and of GFP signal after GFP-NLS (10 μM) delivery (bottom panels). (A) Different concentrations of 6His-CM18-PTD4 (10 μM) and GFP-NLS (10 μM) were co-incubated for 15 secs with THP-1 cells and flow cytometry analysis was performed 4 hours after GFP-NLS delivery. GFP positive cells are counted on the right of the red line threshold and cells without fluorescence are counted on the left. (B) 6His-CM18-PTD4 (10 μM) and GFP-NLS (10 μM) were co-incubated for 15 secs to 120 secs with THP-1 cells and flow cytometry analysis was performed 4 hours after GFP-NLS delivery. (C) Fluorescence microscopy analysis of THP1 cells exposed to GFP-NLS (10 μM) alone. Cell confluence and morphology are shown with bright field view (left panel) and the absence of GFP signal was observed by fluorescence (Scale bars: 50 μm). (D) 6His-CM18-PTD4 (0.5 μM) and GFP-NLS (5 μM) were continuously co-incubated with THP-1 cells in medium with serum and harvested after 1 or 3 days. (E) 6His-CM18-PTD4 (0.8 μM) and GFP-NLS (1 μM) were continuously co-incubated with THP-1 cells in medium with serum and harvested after 1 or 3 days. For 3 days condition, fresh mix containing the peptide and GFP-NLS was added one time daily. (F) Fluorescent microscopy analysis of HeLa cells after a one-minute co-incubation with 6His-CM18-PTD4 (10 μM) and HoxB4 (7 μM). Cells were fixed and permeabilized prior to immuno-labelling with an anti-HoxB4 antibody and a fluorescent secondary antibody 10, 30 and 60 min after the HoxB4 delivery (Scale bars: 50 μm). (G) Real-time PCR analysis of the EGR1 gene expression after co-incubation for 2.5 hours of different concentrations of 6His-CM18-PTD4 (0.5, 0.75, 0.8, 0.9 and 1 μM) and HoxB4 (0.3, 0.9 and 1.5 μM) with THP1 cells in medium with serum.
(TIF)

S3 Fig Nuclear localization of Cas9-NLS and Cpf1-NLS proteins after 6His-CM18-PTD4-mediated delivery in HeLa cells. 6His-CM18-PTD4 (10 μM) and CRISPR Cas9-NLS or CRISPR Cpf1-NLS RNPs were co-incubated for 1 min with HeLa cells. Cells were fixed and permeabilized prior to immuno-labelling of Cas9 or Cpf1 with specific antibodies. Numerical values were obtained comparing nuclei with immuno-labelling signal and hoestch staining. Counting was performed with the ImageJ software https://imagej.nih.gov/ij/.
(TIF)

S4 Fig 6His-CM18-PTD4 mediates endosomal membrane disruption at low concentration. (A) Flow cytometry raw data of HeLa cell viability depending size and coarseness (upper panels) and of GFP signal after GFP-NLS (10 μM) delivery (bottom panels). 6His-CM18-PTD4 (10 μM) and GFP-NLS (10 μM) were co-incubated with HeLa cells for 1 min at 37°C and 4°C or in presence of endocytosis inhibitors. Flow cytometry analysis was performed 4 hours after GFP-NLS delivery. GFP positive cells are counted on the right of the red line threshold and cells without fluorescence are counted on the left. (B) Bright field microscopy analysis shows the confluence and the morphology of HeLa cells co-incubated at 4°C and 37°C for 30 min with different concentrations of 6His-CM18-PTD4 (5 μM, 2.5 μM and 0.5 μM) and FITC-Dextran (10 kDa) (Scale bars: 20 μm). (C) Fluorescence microscopy analysis of 4 randomly chosen areas in the same 96-well pit containing HeLa cells incubated at 37°C for 30 min with 6His-CM18-PTD4 (0.5 μM) and FITC-Dextran (10 kDa). Bright field views (upper panels) shows the confluence and the morphology of HeLa cells and fluorescence microscopy images show the cytosolic and endosomal signal of FITC-Dextran (10 kDa) (bottom panels) (Scale bars: 20 μm).
(TIF)

S5 Fig Correlation between amphiphilic properties of 6His-CM18-PTD4 and protein delivery efficiency. (A) Helical wheel projections of the PTD4, CM18, CM18-PTD4 and 6His-CM18-PTD4 peptides (Top view) were determined and adapted to this article with the free http://rzlab.ucr.edu/scripts/wheel/wheel.cgi software source code. This software measures the hydrophobic moment (μH) of each peptide and the repartition of hydrophobic and hydrophilic amino acid residues along the helical axis. Amino acids are connected with black line respecting the rotation angle of the helix (3.6 residues per tour). On the right of wheel projections, 3D helical structures were built with PyMOL based on the Psipred secondary structure predictions as in S6 Table. Wheel projections and helical structures share the same color code depending the physico-chemical properties of amino acid. Hydrophobic residues are gray, hydrophilic cationic residues are red and neutral residues are green. (B) Correlation between the GFP-NLS transduction efficiency score of each peptide variant and their respective hydrophobic moment. We determined a hydrophobic moment threshold of approximately 5 (dashed line) above which peptide analogues enabled GFP-NLS delivery in HeLa cells.
(TIF)

S1 List Reagents list. (DOCX)
