# Supplementary material for: Membrane permeabilizing amphiphilic peptide delivers recombinant transcription factor and CRISPR-Cas9/Cpf1 ribonucleoproteins in hard-to-modify cells
Source: PLoS One. 2018 Apr 4;13(4):e0195558. doi: 10.1371/journal.pone.0195558 (PMC5884575; doi:10.1371/journal.pone.0195558)
Supplement: S1 Table — (DOCX) [file pone.0195558.s001.docx]

**S1 Table - Peptide sequences and delivery efficiency**

| Domain(s) | Peptide or Shuttle agent | Amino acid (a.a.) sequence | a.a. | MW (kDa) | p.I. | Net  Charge | Hydro-phobic moment  (µ_H_) | Delivery efficiency  (Mean ± SD) (%) | Cell viability  (Mean ± SD) (%) | Score  (delivery / viability) |
| --- | --- | --- | --- | --- | --- | --- | --- | --- | --- | --- |
| ELD | **CM18** | **KWKLFKKIGAVLKVLTTG** | 18 | 2.03 | 10.60 | +5 | 4.28 | 12.9 ± 1.3 | 85.1 ± 1.2 | 1.02 |
| CPD | **PTD4** | **YARAAARQARA** | 11 | 1.2 | 11.72 | +3 | 2.44 | 1.1 ± 0.16 | 94 ± 4.5 | 0.9 |
| ELD-CPD | **CM18-PTD4** | **KWKLFKKIGAVLKVLTTGYARAAARQARA** | 29 | 3.217 | 11.76 | +8 | 6.72 | 57.3 ± 5.3 | 40.3 ± 3.1 | 2.31 |
|  | **3His-CM18-PTD4** | **HHHKWKLFKKIGAVLKVLTTG**  **YARAAARQARA** | 32 | 3.63 | 11.76 | +8 | 7.21 | 39.4 ± 0.5 | 39.2 ± 3.3 | 1.48 |
|  | **6His-CM18-PTD4** | **HHHHHHKWKLFKKIGAVLKVLTTGYARAAARQARA** | 35 | 4.039 | 11.76 | +8 | 7.79 | 64.3 ± 3.2 | 88.6 ± 4.5 | 5.8 |
|  | **9His-CM18-PTD4** | **HHHHHHHHHKWKLFKKIGAVLKVLTTGYARAAARQARA** | 38 | 4.45 | 11.76 | +8 | 7.92 | 36.7 ± 3.3 | 38.7 ± 3.1 | 1.37 |
|  | **12His-CM18-PTD4** | **HHHHHHHHHHHHKWKLFKKIGAVLKVLTTGYARAAARQARA** | 41 | 4.86 | 11.76 | +8 | 7.48 | 36.9 ± 4.3 | 33.4 ± 4.3 | 1.26 |
|  | **CM18-PTD4-6His** | **KWKLFKKIGAVLKVLTTGYARAAARQARAHHHHHH** | 35 | 4.039 | 11.76 | +8 | 6.13 | 61.7 ± 1.8 | 57.7 ± 4.2 | 3.56 |
|  | **CM18-6His-PTD4** | **KWKLFKKIGAVLKVLTTGHHHHHHYARAAARQARA** | 35 | 4.04 | 11.76 | +8 | 5.28 | 44.7 ± 1.5 | 63.9 ± 1.1 | 2.85 |
|  | **6His-CM18-PTD4-6His** | **HHHHHHKWKLFKKIGAVLKVLTTGYARAAARQARAHHHHHH** | 41 | 4.86 | 11.76 | +8 | 7.5 | 62 ± 6 | 88.3 ± 4.1 | 5.45 |
|  | **PTD4-CM18** | **YARAAARQARAKWKLFKKIGAVLKVLTTG** | 29 | 3.217 | 11.76 | +8 | 6.66 | 47.6 ± 2.6 | 33.9 ± 3.7 | 1.61 |
|  | **6His-PTD4-CM18** | **HHHHHHYARAAARQARAKWKLFKKIGAVLKVLTTG** | 35 | 4.039 | 11.76 | +8 | 7.66 | 53.7 ± 4.9 | 83.5 ± 5.7 | 4.5 |

Results computed using the ProtParam^TM^ online tool available from ExPASy^TM^ Bioinformatics Resource Portal (http://web.expasy.org/cgi-bin/protparam/protparam)

MW: Molecular weight

pI: Isoelectric point

Net Charge: Sum of positive (+) and negative (-) charges in the sequence

GFP-NLS (34.8 kDa) delivery efficiency and cell viability were converted in a single numerical value named ‘’Score’’. The score was calculated in a scale with a maximum of 10 by multiplying the rate of GFP positive cells (%) with corresponding cell viability (%).
