# Supplementary material for: Membrane permeabilizing amphiphilic peptide delivers recombinant transcription factor and CRISPR-Cas9/Cpf1 ribonucleoproteins in hard-to-modify cells
Source: PLoS One. 2018 Apr 4;13(4):e0195558. doi: 10.1371/journal.pone.0195558 (PMC5884575; doi:10.1371/journal.pone.0195558)
Supplement: S2 Table — (DOCX) [file pone.0195558.s002.docx]

**S2 Table - Recombinant protein sequences**

| **Protein** | **Recombinant protein sequences**  **(NLS sequence is underlined)** | **p.I.** | **MW**  **(KDa)** |
| --- | --- | --- | --- |
| GFP-NLS  (Feldan Therapeutics engineering) | MHHHHHHGGGGSGGGGSGGASTGTGIRMVSKGEELFTGVVPILVELDGDVNGHKFSVSGEGEGDATYGKLTLKFICTTGKLPVPWPTLVTTLTYGVQCFSRYPDHMKQHDFFKSAMPEGYVQERTIFFKDDGNYKTRAEVKFEGDTLVNRIELKGIDFKEDGNILGHKLEYNYNSHNVYIMADKQKNGIKVNFKIRHNIEDGSVQLADHYQQNTPIGDGPVLLPDNHYLSTQSALSKDPNEKRDHMVLLEFVTAAGITLGMDELYKGGSGGGSGGGSGWIRASSGGRSSDDEATADSQHAAPPKKKRKVGGSGGGSGGGSGGGRGTEIS | 6.46 | 34.85 |
| HoxB4  (Feldan Therapeutics engineering) | MHHHHHHMAMSSFLINSNYVDPKFPPCEEYSQSDYLPSDHSPGYYAGGQRRESSFQPEAGFGRRAACTVQRYAACRDPGPPPPPPPPPPPPPPPGLSPRAPAPPPAGALLPEPGQRCEAVSSSPPPPPCAQNPLHPSPSHSCKEPVVYPWMRKVHVSTVNPNYAGGEPKRSRTAYTRQQVLELEKEFHYNRYLTRRRRVEIAHALCLSERQIKIWFQNRRMKWKKDHKLPNTKIRSGGAAGSAGGPPGR PNGGPRAL | 9.89 | 28.54 |
| Cas9-NLS  (Feldan Therapeutics  #A034a) | MDKKYSIGLDIGTNSVGWAVITDEYKVPSKKFKVLGNTDRHSIKKNLIGALLFDSGETAEATRLKRTARRRYTRRKNRICYLQEIFSNEMAKVDDSFFHRLEESFLVEEDKKHERHPIFGNIVDEVAYHEKYPTIYHLRKKLVDSTDKADLRLIYLALAHMIKFRGHFLIEGDLNPDNSDVDKLFIQLVQTYNQLFEENPINASGVDAKAILSARLSKSRRLENLIAQLPGEKKNGLFGNLIALSLGLTPNFKSNFDLAEDAKLQLSKDTYDDDLDNLLAQIGDQYADLFLAAKNLSDAILLSDILRVNTEITKAPLSASMIKRYDEHHQDLTLLKALVRQQLPEKYKEIFFDQSKNGYAGYIDGGASQEEFYKFIKPILEKMDGTEELLVKLNREDLLRKQRTFDNGSIPHQIHLGELHAILRRQEDFYPFLKDNREKIEKILTFRIPYYVGPLARGNSRFAWMTRKSEETITPWNFEEVVDKGASAQSFIERMTNFDKNLPNEKVLPKHSLLYEYFTVYNELTKVKYVTEGMRKPAFLSGEQKKAIVDLLFKTNRKVTVKQLKEDYFKKIECFDSVEISGVEDRFNASLGTYHDLLKIIKDKDFLDNEENEDILEDIVLTLTLFEDREMIEERLKTYAHLFDDKVMKQLKRRRYTGWGRLSRKLINGIRDKQSGKTILDFLKSDGFANRNFMQLIHDDSLTFKEDIQKAQVSGQGDSLHEHIANLAGSPAIKKGILQTVKVVDELVKVMGRHKPENIVIEMARENQTTQKGQKNSRERMKRIEEGIKELGSQILKEHPVENTQLQNEKLYLYYLQNGRDMYVDQELDINRLSDYDVDHIVPQSFLKDDSIDNKVLTRSDKNRGKSDNVPSEEVVKKMKNYWRQLLNAKLITQRKFDNLTKAERGGLSELDKAGFIKRQLVETRQITKHVAQILDSRMNTKYDENDKLIREVKVITLKSKLVSDFRKDFQFYKVREINNYHHAHDAYLNAVVGTALIKKYPKLESEFVYGDYKVYDVRKMIAKSEQEIGKATAKYFFYSNIMNFFKTEITLANGEIRKRPLIETNGETGEIVWDKGRDFATVRKVLSMPQVNIVKKTEVQTGGFSKESILPKRNSDKLIARKKDWDPKKYGGFDSPTVAYSVLVVAKVEKGKSKKLKSVKELLGITIMERSSFEKNPIDFLEAKGYKEVKKDLIIKLPKYSLFELENGRKRMLASAGELQKGNELALPSKYVNFLYLASHYEKLKGSPEDNEQKQLFVEQHKHYLDEIIEQISEFSKRVILADANLDKVLSAYNKHRDKPIREQAENIIHLFTLTNLGAPAAFKYFDTTIDRKRYTSTKEVLDATLIHQSITGLYETRIDLSQLGGDGGRSSDDEATADSQHAAPPKKKRKVGGSGGGSGGGSGGGRHHHHHH | 9.05 | 162.9 |
| Cpf1-NLS  (Feldan Therapeutics  #A037a) | MTQFEGFTNLYQVSKTLRFELIPQGKTLKHIQEQGFIEEDKARNDHYKELKPIIDRIYKTYADQCLQLVQLDWENLSAAIDSYRKEKTEETRNALIEEQATYRNAIHDYFIGRTDNLTDAINKRHAEIYKGLFKAELFNGKVLKQLGTVTTTEHENALLRSFDKFTTYFSGFYENRKNVFSAEDISTAIPHRIVQDNFPKFKENCHIFTRLITAVPSLREHFENVKKAIGIFVSTSIEEVFSFPFYNQLLTQTQIDLYNQLLGGISREAGTEKIKGLNEVLNLAIQKNDETAHIIASLPHRFIPLFKQILSDRNTLSFILEEFKSDEEVIQSFCKYKTLLRNENVLETAEALFNELNSIDLTHIFISHKKLETISSALCDHWDTLRNALYERRISELTGKITKSAKEKVQRSLKHEDINLQEIISAAGKELSEAFKQKTSEILSHAHAALDQPLPTTLKKQEEKEILKSQLDSLLGLYHLLDWFAVDESNEVDPEFSARLTGIKLEMEPSLSFYNKARNYATKKPYSVEKFKLNFQMPTLASGWDVNKEKNNGAILFVKNGLYYLGIMPKQKGRYKALSFEPTEKTSEGFDKMYYDYFPDAAKMIPKCSTQLKAVTAHFQTHTTPILLSNNFIEPLEITKEIYDLNNPEKEPKKFQTAYAKKTGDQKGYREALCKWIDFTRDFLSKYTKTTSIDLSSLRPSSQYKDLGEYYAELNPLLYHISFQRIAEKEIMDAVETGKLYLFQIYNKDFAKGHHGKPNLHTLYWTGLFSPENLAKTSIKLNGQAELFYRPKSRMKRMAHRLGEKMLNKKLKDQKTPIPDTLYQELYDYVNHRLSHDLSDEARALLPNVITKEVSHEIIKDRRFTSDKFFFHVPITLNYQAANSPSKFNQRVNAYLKEHPETPIIGIDRGERNLIYITVIDSTGKILEQRSLNTIQQFDYQKKLDNREKERVAARQAWSVVGTIKDLKQGYLSQVIHEIVDLMIHYQAVVVLENLNFGFKSKRTGIAEKAVYQQFEKMLIDKLNCLVLKDYPAEKVGGVLNPYQLTDQFTSFAKMGTQSGFLFYVPAPYTSKIDPLTGFVDPFVWKTIKNHESRKHFLEGFDFLHYDVKTGDFILHFKMNRNLSFQRGLPGFMPAWDIVFEKNETQFDAKGTPFIAGKRIVPVIENHRFTGRYRDLYPANELIALLEEKGIVFRDGSNILPKLLENDDSHAIDTMVALIRSVLQMRNSNAATGEDYINSPVRDLNGVCFDSRFQNPEWPMDADANGAYHIALKGQLLLNHLKESKDLKLQNGISNQDWLAYIQELRNGGRSSDDEATADSQHAAPPKKKRKVGGSGGGSGGGSGGGRHHHHHH | 8.34 | 155.7 |
