# Supplementary material for: Membrane permeabilizing amphiphilic peptide delivers recombinant transcription factor and CRISPR-Cas9/Cpf1 ribonucleoproteins in hard-to-modify cells
Source: PLoS One. 2018 Apr 4;13(4):e0195558. doi: 10.1371/journal.pone.0195558 (PMC5884575; doi:10.1371/journal.pone.0195558)
Supplement: S3 Table — (DOCX) [file pone.0195558.s003.docx]

**S3 Table - List of cell types**

| Cell lines | Description | ATCC/others | Culture media | Serum | Additives |
| --- | --- | --- | --- | --- | --- |
| HeLa  (adherent cells) | Human cervical carcinoma cells | ATCC^TM^ CCL-2 | DMEM | 10% FBS | L-glutamine 2 mM  Penicillin 100 units  Streptomycin 100µg/mL |
| HEK 293T  (adherent cells) | Human embryonic Epithelial kidney cells | ATCC^TM^ CRL-3216 | DMEM | 10% FBS | L-glutamine 2 mM  Penicillin 100 units  Streptomycin 100µg/mL |
| CHO  (adherent cells) | Chinese hamster ovary cells | ATCC^TM^ CCL-61 | DMEM | 10% FBS | L-glutamine 2 mM  Penicillin 100 units  Streptomycin 100 µg/mL |
| NIH3T3  (adherent cells) | Mouse fibroblasts | ATCC^TM^ CRL-1658 | DMEM | 10% Calf serum | L-glutamine 2 mM  Penicillin 100 units  Streptomycin 100 µg/mL |
| Balb3T3  (adherent cells) | Mouse fibroblasts | ATCC^TM^ CCL-163 | DMEM | 10% Calf serum | L-glutamine 2 mM  Penicillin 100 units  Streptomycin 100 µg/mL |
| THP-1  (suspension cells) | Acute human monocytic leukemia | ATCC^TM^ TIB202 | RPMI 1640 | 10% FBS | 2- mercaptoethanol 0.05 mM  L-glutamine 2 mM  Penicillin 100 units  Streptomycin 100µg/mL |
| Jurkat  (suspension cells) | Human T cells | ATCC^TM^ TIB-152 | RPMI 1640 | 10% FBS | L-glutamine 2 mM  Penicillin 100 units  Streptomycin 100 µg/mL |
| HT2  (suspension cells) | Mouse T lymphocytes | ATCC^TM^ CRL-1841 | RPMI 1640 | 10% FBS | 200 IU/mL IL-2  β-mercaptoethanol 0.05mM  L-glutamine 2 mM  Penicillin 100 units  Streptomycin 100 µg/mL |
| CA46  (suspension cells) | Homo sapiens Burkitt's lymphoma | ATCC^TM^ CRL-1648 | RPMI 1640 | 20% FBS | L-glutamine 2 mM  Penicillin 100 units  Streptomycin 100 µg/mL |
| NK  (suspension cells) | Human normal Peripheral Blood CD56+ lymphocyte | All cells^TM^  #PB012-PF | RPMI 1640 | 10% FBS | 200 IU/mL IL-2  L-glutamine 2 mM  Penicillin 100 units  Streptomycin 100 µg/mL |
| HCC-78  (adherent cells) | Human adenocarcinoma lung cell | Gift from  Horizon Inc. | RPMI 1640 | 20% FBS | L-glutamine 2 mM  Penicillin 100 units  Streptomycin 100 µg/mL |
| NIC-H196  (adherent cells) | Human small cell lung cancer | Gift from  Horizon Inc. | RPMI 1640 | 10% FBS | L-glutamine 2 mM  Penicillin 100 units  Streptomycin 100 µg/mL |
| DOHH2  (suspension cells) | Human B cell lymphoma | Gift from  Horizon Inc. | RPMI 1640 | 10% FBS | L-glutamine 2 mM  Penicillin 100 units  Streptomycin 100 µg/mL |
| KMS  (suspension cells) | Myeloma bone marrow | Gift from  Horizon Inc. | Advanced  RPMI 1640 | 10% FBS | L-glutamine 2 mM  Penicillin 100 units  Streptomycin 100 µg/mL |
| REC-1  (suspension cells) | human lymph node mantel cell | Gift from  Horizon Inc. | RPMI 1640 | 10% FBS | L-glutamine 2 mM  Penicillin 100 units  Streptomycin 100 µg/mL |
| Myoblasts  (primary adherent cells) | Human myoblasts | Kindly provided by Professor JP Tremblay | MB1 | 15% FBS | ITS 1x, FGF 2 10 ng/mL,  Dexamethasone 0.39µg/mL,  BSA 0.5mg/mL,  MB1 85% |
| MSC (adherent cells) | Mesenchymal stem cells | Assays performed by CCRM | Mesencult MSC medium | 2% FBS | L-glutamine 2 mM |
| ESC (adherent cells) | Epithelial stem cells | Assays performed by CCRM | DMEM/F12  Life Tech. | 10% FBS | L-glutamine 2 mM  HEPES |
| CD34+ (suspension cells) | Hematopoïetic stem cells | Assays performed by Hôpital Rosemont | RPMI advanced  Thermofisher | 10% FBS | 200 IU/mL IL-2  L-glutamine 2 mM  Penicillin 100 units  Streptomycin 100 µg/mL |
