# Supplementary material for: Membrane permeabilizing amphiphilic peptide delivers recombinant transcription factor and CRISPR-Cas9/Cpf1 ribonucleoproteins in hard-to-modify cells
Source: PLoS One. 2018 Apr 4;13(4):e0195558. doi: 10.1371/journal.pone.0195558 (PMC5884575; doi:10.1371/journal.pone.0195558)
Supplement: S4 Table — (DOCX) [file pone.0195558.s004.docx]

**S4 Table - List of gene targets and guide RNAs**

| **Gene targets and**  **guide RNAs** | **Sequences of oligonucleotides** |
| --- | --- |
| B2M gene | GGGAGGAACTTCTTGGCACAGAACTTTCCAAACACTTTTTCCTGAAGGGATACAAGAAGCAAGAAAGGTACTCTTTCACTAGGACCTTCTCTGAGCTGTCCTCAGGATGCTTTTGGGACTATTTTTCTTACCCAGAGAATGGAGAAACCCTGCAGGGAATTCCCAAGCTGTAGTTATAAACAGAAGTTCTCCTTCTGCTAGGTAGCATTCAAAGATCTTAATCTTCTGGGTTTCCGTTTTCTCGAATGAAAAATGCAGGTCCGAGCAGTTAACTGGCTGGGGCACCATTAGCAAGTCACTTAGCATCTCTGGGGCCAGTCTGCAAAGCGAGGGGGCAGCCTTAATGTGCCTCCAGCCTGAAGTCCTAGAATGAGCGCCCGGTGTCCCAAGCTGGGGCGCGCACCCCAGATCGGAGGGCGCCGATGTACAGACAGCAAACTCACCCAGTCTAGTGCATGCCTTCTTAAACATCACGAGACTCTAAGAAAAGGAAACTGAAAACGGGAAAGTCCCTCTCTCTAACCTGGCACTGCGTCGCTGGCTTGGAGACAGGTGACGGTCCCTGCGGGCCTTGTCCTGATTGGCTGGGCACGCGTTTAATATAAGTGGAGGCGTCGCGCTGGCGGGCATTCCTGAAGCTGACAGCATTCGGGCCGAGATGTCTCGCTCCGTGGCCTTAGCTGTGCTCGCGCTACTCTCTCTTTCTGGCCTGGAGGCTATCCAGCGTGAGTCTCTCCTACCCTCCCGCTCTGGTCCTTCCTCTCCCGCTCTGCACCCTCTGTGGCCCTCGCTGTGCTCTCTCGCTCCGTGACTTCCCTTCTCCAAGTTCTCCTTGGTGGCCCGCCGTGGGGCTAGTCCAGGGCTGGATCTCGGGGAAGCGGCGGGGTGGCCTGGGAGTGGGGAAGGGGGTGCGCACCCGGGACGCGCGCTACTTGCCCCTTTCGGCGGGGAGCAGGGGAGACCTTTGGCCTACGGCGACGGGAGGGTCGGGACAAAGTTTAGGGCGTCGATAAGCGTC |
| tracrRNA (Cas9 RNP complex) | 5′-AAACAGCAUAGCAAGUUAAAAUAAGGCUAGUCCGUUAUCAACUUGAAAAAGUGGCACCGAGUCGGUGCU-3′ |
| crRNA-1 flanking B2M gene (exon1) | 5′- AAUUUCUACUGUUGUAGAUAUAUAAGUGGAGGCGUCGCG-3′ |
| crRNA-2 flanking B2M gene (exon2) | 5′- AAUUUCUACUGUUGUAGAUAUCCAUCCGACAUUGAAGUU-3′ |
| crRNA-3 flanking B2M gene (exon2) | 5′- AAUUUCUACUCUUGUAGAUCCGAUAUUCCUCAGGUACUCCA-3′ |
| HPRT gene | ACACATCCATGGGACTTCTGCCTCCAGAGTAAGAGGCAGGTATTCAAAGGACAGTGAATTAAAAGACTAAACTCGATCATTTATTTCTTACTTATGACCACATTTATCATCTGAAATAATAATGCAACAATTATTGTATATTAAAGCTGTTCAACTATTTCAGCCAACAAGAAGTGTCACCCTAGCCTGGCCAGGTTCCAGTTCTAAGGACGTCTGTACTAGACTACAGCTTTATGTGACTAATGGGAACCATCAGTCTGTTCAAATTATGAGGTGCTGGAAGGAGAAAACAATTCTCTTTCCTAAATTTTTATGCGTGTTTTGAAAAATGAGTGAGAAAAAGAAGCAATTACTTACATTCAAATCCCTGAAGTATTCATTATAGTCAAGGGCATATCCTACAACAAACTTGTCTGGAATTTCAAATCCAACAACTAAAAAGAATCATAATTCATCATTTAGATAAAGAAAACATCACTTTTAAATCTAATACTGGCAAATGTGCCTCTCTACAAATATTCTCTAAGCAATTATAAGCCATTTCACATAAAACTCTTTTAGGTTAAAGATGGTTAAATGATTGACAAAAAAAGTAATTCACTTACAGTCTGGCTTATATCCAACACTTCGTGGGGTCCTTTTCACCAGCAAGCTGTTAATTACAAAATGTGACATATAACATACAGAGAGACTACAGGGCATTACAAAAGAGAAGACTGACGTTTCTAAACACTGTTTCATTTCATCCGTGCTGAGTGTACCATGGTCACTTTTAACACACCCAAGGAAAGACTATGAAATGGAGAGCTAAATTATGGGGATTACTAGGAAGGGGCAGCAATGAGTTGACACTACAGACAAGGCACTTGGTTGATCACCTGGAACCTGAAGGACAGTTCTGAGACCTGCACCCTGACTACCCATGTGTCCATTGAAGGGGAGCTAATAAGGAGGATTAATGGGTACAGAGTGTCAATGAGCAAAGATGAAAAGGGCTCTCAGTCTAGAAACCTTGGAAATACAATCCCTAAACTATAAAAGGGTGTGGGAGAGGTGAGCATCACCTTTTATCACAACATTCTT |
| crRNA flanking HPRT gene | 5’-AATTATGGGGATTACTAGGAGTTTTAGAGCTATGCT-3’ |
| DNMT1 gene | CTGGGACTCAGGCGGGTCACCTACCCACGTTCGTGGCCCCATCTTTCTCAAGGGGCTGCTGTGAGGATTGAGTGAGTTGCACGTGTCAAGTGCTTAGAGCAGGCGTGCTGCACACAGCAGGCCTTTGGTCAGGTTGGCTGCTGGGCTGGCCCTGGGGCCGTTTCCCTCACTCCTGCTCGGTGAATTTGGCTCAGCAGGCACCTGCCTCAGCTGCTCACTTGAGCCTCTGGGTCTAGAACCCTCTGGGGACCGTTTGAGGAGTGTTCAGTCTCCGTGAACGTTCCCTTAGCACTCTGCCACTTATTGGGTCAGCTGTTAACATCAGTACGTTAATGTTTCCTGATGGTCCATGTCTGTTACTCGCCTGTCAAGTGGCGTGACACCGGGCGTGTTCCCCAGAGTGACTTTTCCTTTTATTTCCCTTCAGCTAAAATAAAGGAGGAGGAAGCTGCTAAGGACTAGTTCTGCCCTCCCGTCACCCCTGTTTCTGGCACCAGGAATCCCCAACATGCACTGATGTTGTGTTTTTAACATGTCAATCTGTCCGTTCACATGTGTGGTACATGGTGTTTGTGGCCTTGGCTGACATGAAGCTGTTGTGTGAGG |
| crRNA flanking DNMT1 gene | 5’-AAUUUCUACUGUUGUAGAUCUGAUGGUCCAUGUCUGUUACUC-3’ |
| Short DNA template (insertion in HPRT gene) | 5’-TGAAATGGAGAGCTAAATTATGGGGATTACAAGCTTGATAGC  GAAGGGGCAGCAATGAGTTGACACTACAGA-3’ |
| EcoR1 DNA template (insertion in DNMT1 gene) | 5’-AGTACGTTAATGTTTCCTGATGGTCCATGTCTG  TTGAATTCACTCGCCTGTCAAGTGGCGTGACACCGGGCGTGTT-3’ |
