# Supplementary material for: Membrane permeabilizing amphiphilic peptide delivers recombinant transcription factor and CRISPR-Cas9/Cpf1 ribonucleoproteins in hard-to-modify cells
Source: PLoS One. 2018 Apr 4;13(4):e0195558. doi: 10.1371/journal.pone.0195558 (PMC5884575; doi:10.1371/journal.pone.0195558)
Supplement: S5 Table — (DOCX) [file pone.0195558.s005.docx]

**S5 Table - Cleavage products after CRISPR RNPs delivery**

| **Gene target** | **Gene size (pb)** | **Cleavage product 1 size (pb)** | **Cleavage product 2 size (pb)** |
| --- | --- | --- | --- |
| HPRT  (Hypoxanthine Phosphoribosyl transferase 1) | 1080 | 828 | 255 |
| B2M  (Beta-2-Microglobulin) | 1018 | 680 | 338 |
| DNMT1  (DNA (Cytosine-5)-Methyltransferase 1) | 606 | 358 | 248 |
