# Supplementary material for: Membrane permeabilizing amphiphilic peptide delivers recombinant transcription factor and CRISPR-Cas9/Cpf1 ribonucleoproteins in hard-to-modify cells
Source: PLoS One. 2018 Apr 4;13(4):e0195558. doi: 10.1371/journal.pone.0195558 (PMC5884575; doi:10.1371/journal.pone.0195558)
Supplement: S6 Table — (DOCX) [file pone.0195558.s006.docx]

**S6 Table - Peptide sequences and primary structures**

| **Peptides** | **PSSpred prediction software**  (C = coil, E = Beta sheet,  H = helical structure) | **Psipred prediction software**  (C = coil, H = helical structure) |
| --- | --- | --- |
| PTD4 | YARAAARQARA  HHHHHHHHHHC | YARAAARQARA  CHHHHHHHHHC |
| CM18 | KWKLFKKIGAVLKVLTTG  CHHHHHHHHHHHHHHECC | KWKLFKKIGAVLKVLTTG  CCHHHHHHHHHHHHHHCC |
| CM18-PTD4 | KWKLFKKIGAVLKVLTTGYARAAARQARA  CHHHHHHHHHHHHHHHHHHHHHHHHHHHC | KWKLFKKIGAVLKVLTTGYARAAARQARA  CCHHHHHHHHHHHHHHHHHHHHHHHHHHC |
| 6His-CM18-PTD4 | HHHHHHKWKLFKKIGAVLKVLTTGYARAAARQARA  CCCCHHHHHHHHHHHHHHHHHHHHHHHHHHHHHHC | HHHHHHKWKLFKKIGAVLKVLTTGYARAAARQARA  CCCCHHHHHHHHHHHHHHHHHHHHHHHHHHHHHHC |
