# Supplementary figures and images for: Membrane permeabilizing amphiphilic peptide delivers recombinant transcription factor and CRISPR-Cas9/Cpf1 ribonucleoproteins in hard-to-modify cells
Source: PLoS One. 2018 Apr 4;13(4):e0195558. doi: 10.1371/journal.pone.0195558 (PMC5884575; doi:10.1371/journal.pone.0195558)

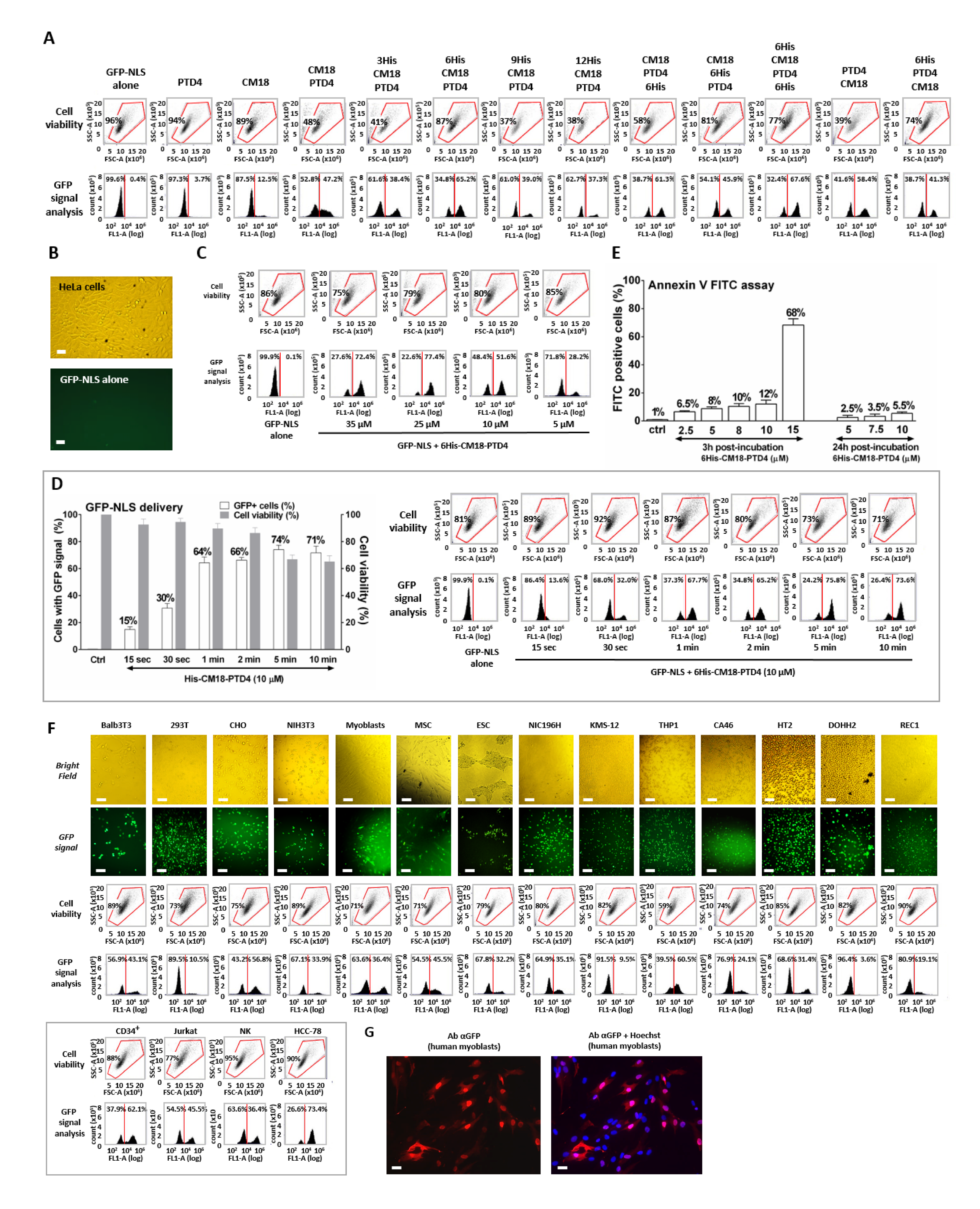

Supplement: S1 Fig — (A) Flow cytometry raw data of HeLa cell viability depending size and coarseness (upper panels) and of GFP signal after GFP-NLS (10 μM) delivery (bottom panels). CM18 /PTD4 peptides analogues (10 μM) and GFP-NLS (10 μM) were co-incubated with HeLa cells for 1 min and flow cytometry analysis was performed 4 hours after GFP-NLS delivery. GFP positive cells are counted on the right of the red line threshold and cells without fluorescence are counted on the left. (B) Microscopy analysis of HeLa cells incubated with GFP-NLS alone. Microscopy images show the HeLa confluence and morphology in bright field (left panel) and the absence of GFP signal (right panel) (Scale bars: 50 μm). (C) Flow cytometry raw data of HeLa cell viability depending size and coarseness (upper panels) and of GFP signal after GFP-NLS (10 μM) delivery (bottom panels). Different concentrations of 6His-CM18-PTD4 (5 μM to 35 μM) and GFP-NLS (10 μM) were co-incubated with HeLa cells for 1 min and flow cytometry analysis was performed 4 hours after GFP-NLS delivery. (D) Flow cytometry analysis of HeLa cells co-incubated for 15 secs to 10 min with 6His-CM18-PTD4 (10 μM) and GFP-NLS (10 μM). Corresponding flow cytometry raw data are shown on the right of the panel. (E) Flow cytometry analysis of HeLa cells exposed to the pre-apoptosis marker FITC-Annexin V 3 h and 24 h after a one-minute incubation with different concentrations of 6His-CM18-PTD4 (2.5 μM to 15 μM). Fluorescence microscopy and flow cytometry analysis of multiple mammalian cell types (Balb3T3, 293T, CHO, NIH3T3, Myoblasts, MSC, ESC, NIC196H, KMS-12, THP1, CA46, HT2, DOHH2, REC-1 cells) incubated with 6His-CM18-PTD4 (10 μM for 1 min in adherent cells; 5 μM for 30 secs in suspension cells) and GFP-NLS (10 μM). Fluorescent microscopy analysis shows bright field views of cells (upper panels) and emanating GFP signal (bottom panels) (Scale bars: 100 μm). Flow cytometry analysis in the grey square relate to fluorescence microscopy images from CD34+, [file pone.0195558.s007.tif]

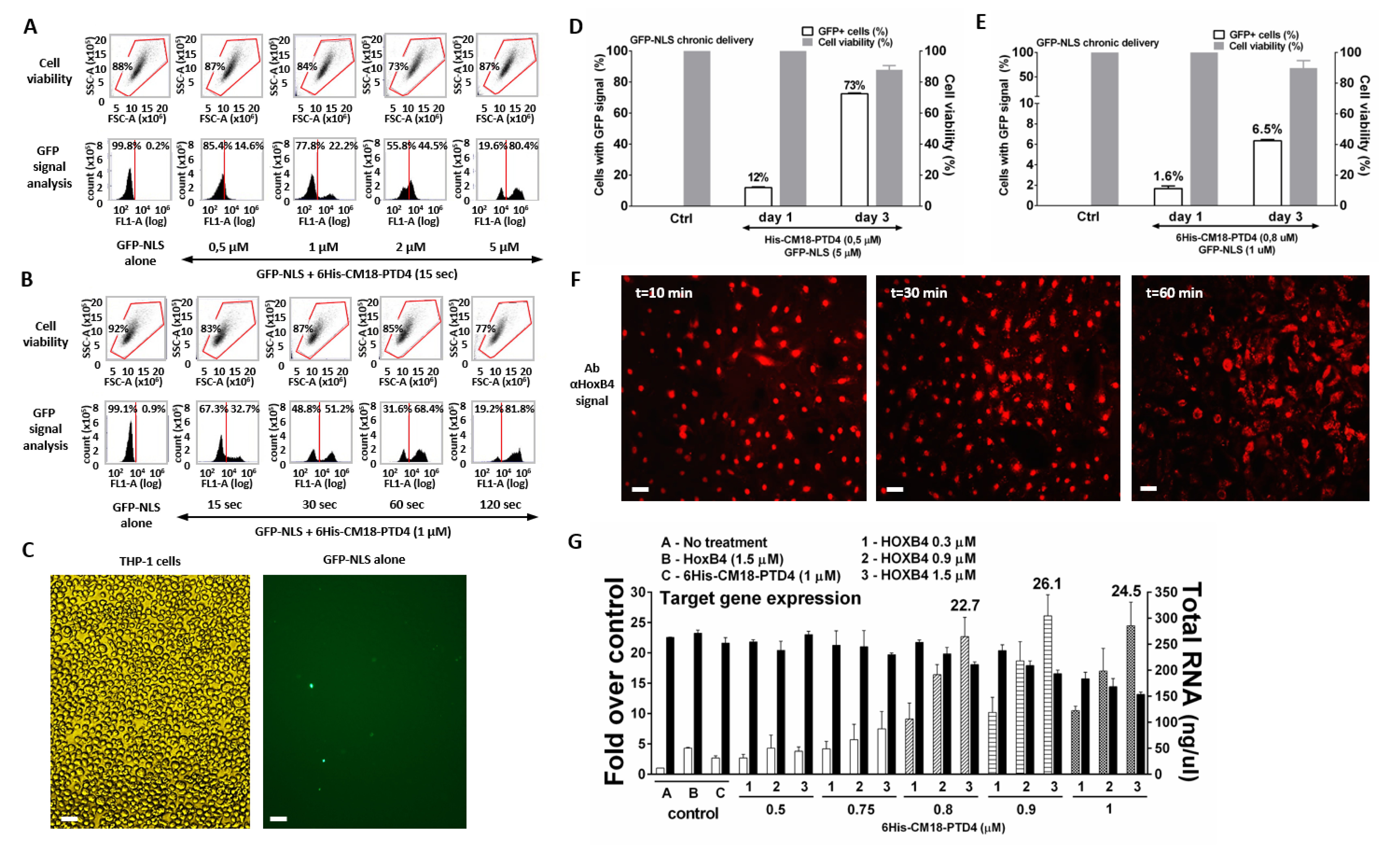

Supplement: S2 Fig — (A-B) Flow cytometry raw data of THP-1 cell viability depending size and coarseness (upper panels) and of GFP signal after GFP-NLS (10 μM) delivery (bottom panels). (A) Different concentrations of 6His-CM18-PTD4 (10 μM) and GFP-NLS (10 μM) were co-incubated for 15 secs with THP-1 cells and flow cytometry analysis was performed 4 hours after GFP-NLS delivery. GFP positive cells are counted on the right of the red line threshold and cells without fluorescence are counted on the left. (B) 6His-CM18-PTD4 (10 μM) and GFP-NLS (10 μM) were co-incubated for 15 secs to 120 secs with THP-1 cells and flow cytometry analysis was performed 4 hours after GFP-NLS delivery. (C) Fluorescence microscopy analysis of THP1 cells exposed to GFP-NLS (10 μM) alone. Cell confluence and morphology are shown with bright field view (left panel) and the absence of GFP signal was observed by fluorescence (Scale bars: 50 μm). (D) 6His-CM18-PTD4 (0.5 μM) and GFP-NLS (5 μM) were continuously co-incubated with THP-1 cells in medium with serum and harvested after 1 or 3 days. (E) 6His-CM18-PTD4 (0.8 μM) and GFP-NLS (1 μM) were continuously co-incubated with THP-1 cells in medium with serum and harvested after 1 or 3 days. For 3 days condition, fresh mix containing the peptide and GFP-NLS was added one time daily. (F) Fluorescent microscopy analysis of HeLa cells after a one-minute co-incubation with 6His-CM18-PTD4 (10 μM) and HoxB4 (7 μM). Cells were fixed and permeabilized prior to immuno-labelling with an anti-HoxB4 antibody and a fluorescent secondary antibody 10, 30 and 60 min after the HoxB4 delivery (Scale bars: 50 μm). (G) Real-time PCR analysis of the EGR1 gene expression after co-incubation for 2.5 hours of different concentrations of 6His-CM18-PTD4 (0.5, 0.75, 0.8, 0.9 and 1 μM) and HoxB4 (0.3, 0.9 and 1.5 μM) with THP1 cells in medium with serum. (TIF) [file pone.0195558.s008.tif]

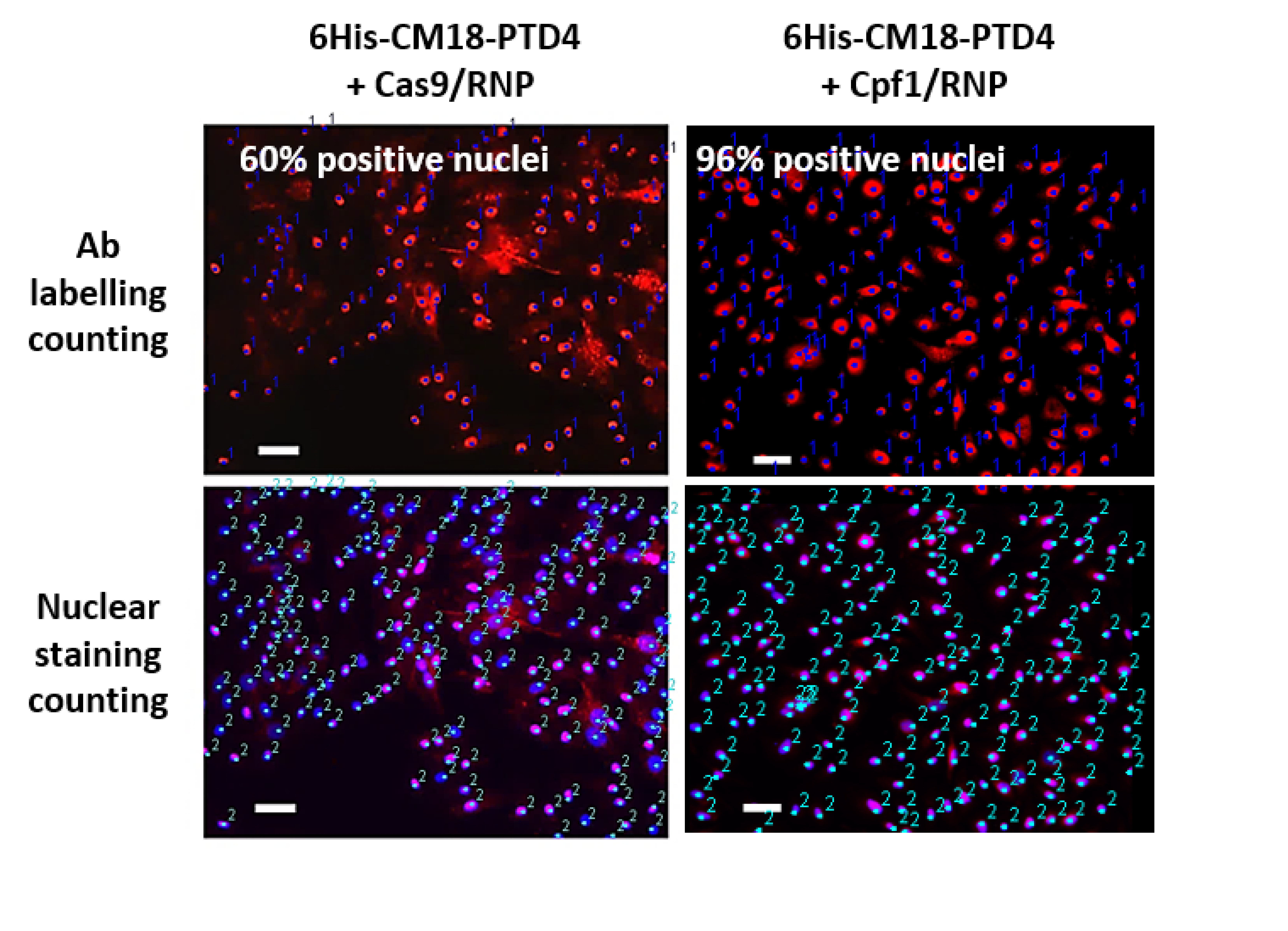

Supplement: S3 Fig — 6His-CM18-PTD4 (10 μM) and CRISPR Cas9-NLS or CRISPR Cpf1-NLS RNPs were co-incubated for 1 min with HeLa cells. Cells were fixed and permeabilized prior to immuno-labelling of Cas9 or Cpf1 with specific antibodies. Numerical values were obtained comparing nuclei with immuno-labelling signal and hoestch staining. Counting was performed with the ImageJ software https://imagej.nih.gov/ij/. (TIF) [file pone.0195558.s009.tif]

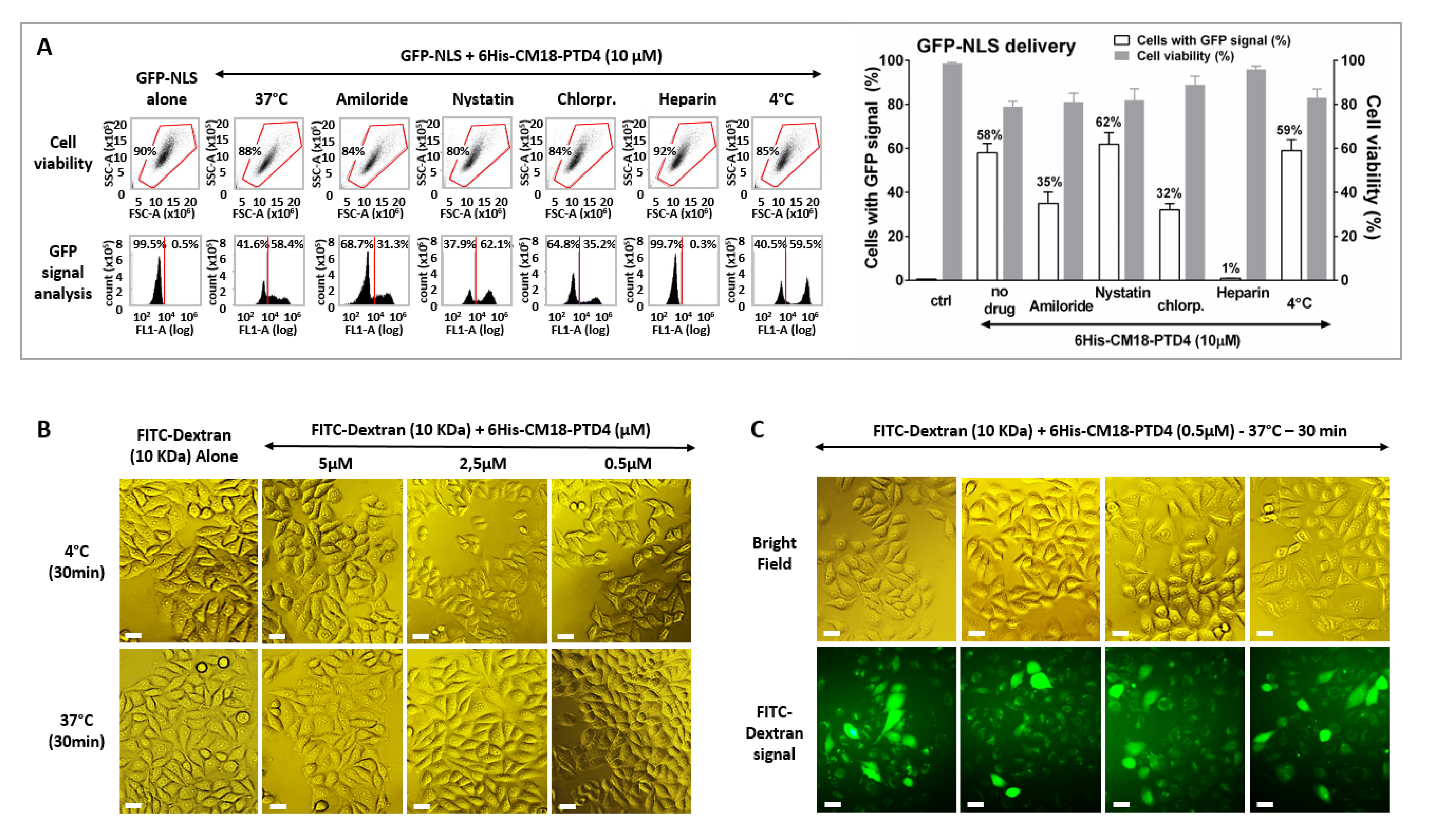

Supplement: S4 Fig — (A) Flow cytometry raw data of HeLa cell viability depending size and coarseness (upper panels) and of GFP signal after GFP-NLS (10 μM) delivery (bottom panels). 6His-CM18-PTD4 (10 μM) and GFP-NLS (10 μM) were co-incubated with HeLa cells for 1 min at 37°C and 4°C or in presence of endocytosis inhibitors. Flow cytometry analysis was performed 4 hours after GFP-NLS delivery. GFP positive cells are counted on the right of the red line threshold and cells without fluorescence are counted on the left. (B) Bright field microscopy analysis shows the confluence and the morphology of HeLa cells co-incubated at 4°C and 37°C for 30 min with different concentrations of 6His-CM18-PTD4 (5 μM, 2.5 μM and 0.5 μM) and FITC-Dextran (10 kDa) (Scale bars: 20 μm). (C) Fluorescence microscopy analysis of 4 randomly chosen areas in the same 96-well pit containing HeLa cells incubated at 37°C for 30 min with 6His-CM18-PTD4 (0.5 μM) and FITC-Dextran (10 kDa). Bright field views (upper panels) shows the confluence and the morphology of HeLa cells and fluorescence microscopy images show the cytosolic and endosomal signal of FITC-Dextran (10 kDa) (bottom panels) (Scale bars: 20 μm). (TIF) [file pone.0195558.s010.tif]

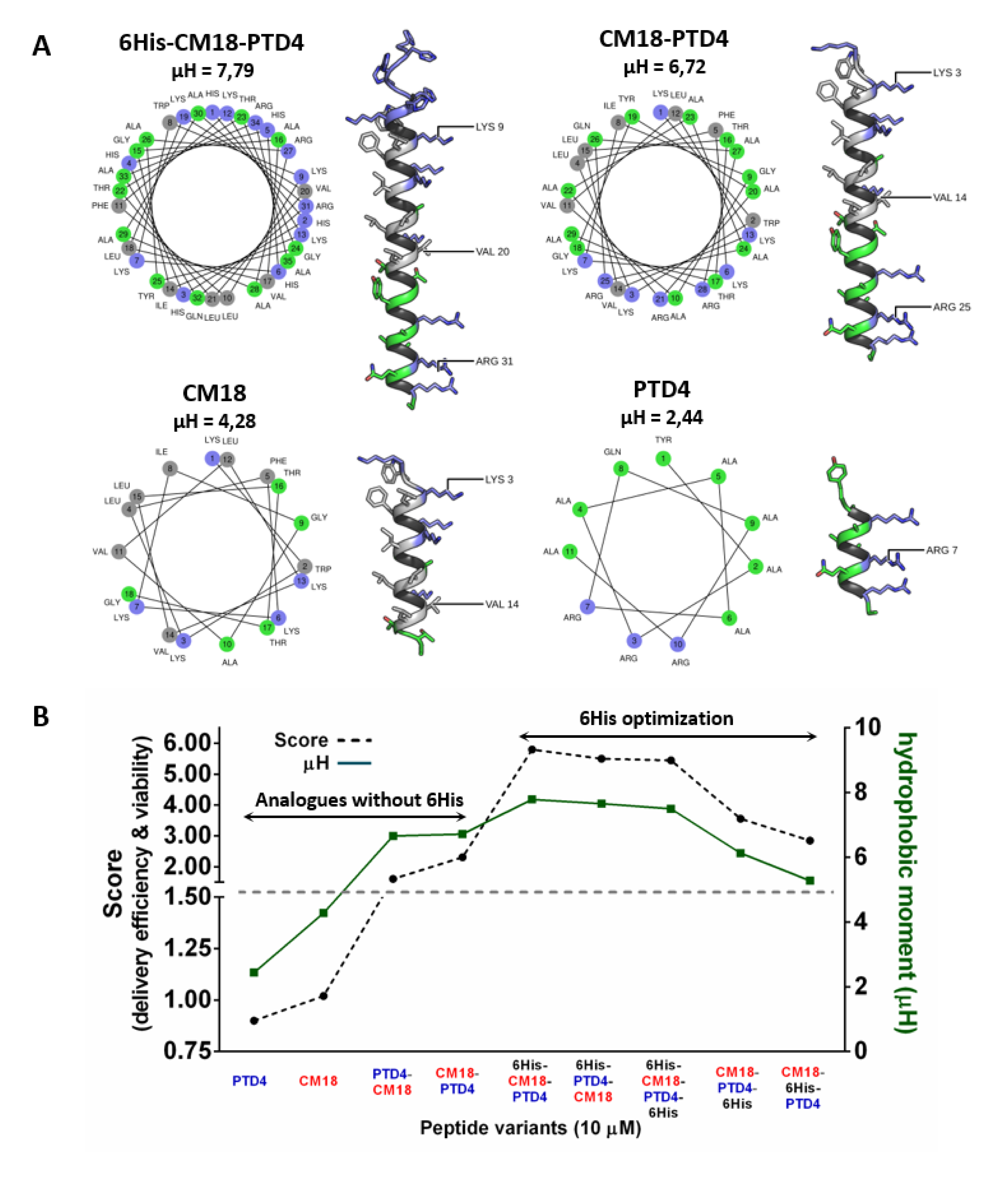

Supplement: S5 Fig — (A) Helical wheel projections of the PTD4, CM18, CM18-PTD4 and 6His-CM18-PTD4 peptides (Top view) were determined and adapted to this article with the free http://rzlab.ucr.edu/scripts/wheel/wheel.cgi software source code. This software measures the hydrophobic moment (μH) of each peptide and the repartition of hydrophobic and hydrophilic amino acid residues along the helical axis. Amino acids are connected with black line respecting the rotation angle of the helix (3.6 residues per tour). On the right of wheel projections, 3D helical structures were built with PyMOL based on the Psipred secondary structure predictions as in S6 Table. Wheel projections and helical structures share the same color code depending the physico-chemical properties of amino acid. Hydrophobic residues are gray, hydrophilic cationic residues are red and neutral residues are green. (B) Correlation between the GFP-NLS transduction efficiency score of each peptide variant and their respective hydrophobic moment. We determined a hydrophobic moment threshold of approximately 5 (dashed line) above which peptide analogues enabled GFP-NLS delivery in HeLa cells. (TIF) [file pone.0195558.s011.tif]
