# Supplementary material for: Membrane permeabilizing amphiphilic peptide delivers recombinant transcription factor and CRISPR-Cas9/Cpf1 ribonucleoproteins in hard-to-modify cells
Source: PLoS One. 2018 Apr 4;13(4):e0195558. doi: 10.1371/journal.pone.0195558 (PMC5884575; doi:10.1371/journal.pone.0195558)
Supplement: S1 List — (DOCX) [file pone.0195558.s012.docx]

**Reagents list - Supporting information**

| **Reagent** | **Supplier** | **Catalog reference** | **Concentration used** |
| --- | --- | --- | --- |
| Annexin V FITC | Invitrogen | A13199 | Dilution 1/ 20 |
| Sytox Red | Invitrogen | S34859 | 5 nM |
| Calcein | Sigma-Aldrich | 14728 | 100 μM |
| Fluorescein isothiocyanate–dextran | Sigma-Aldrich | FD10S | 2.5 mg/ml |
| LysoTracker | Thermo Fisher | Red DND-99 | 50 nM |
| Hoechst | Invitrogen | 33258 | 5 µg/ml |
| Anti-HoxB4 antibody | Novus Bio | NBP2-37257 | Dilution 1/500 |
| Anti-Cas9 antibody | Millipore | MAC133 | Dilution 1/500 |
| Anti-Cpf1 antibody | Agrisera | AS16 3841 | Dilution 1/500 |
| Alexa^TM^-594 | Abcam | 150116 | Dilution 1/1000 |
| CRISPRMax | Thermo Fisher | CMAX00015 | 50 nM |
| RNAimax | Thermo Fisher | 13778030 | 10 nM |
| Dharmafect | Dharmacon | T-2001-01 | 0.4 µg / well (96) |
| Chlorpromazine | Sigma-Aldrich | C8138 | 30 nM |
| Nystatin | Sigma-Aldrich | N6261-5MU | 50 µg/ml |
| Amiloride | Sigma-Aldrich | A-7410 | 1 mM |
| Heparin | Sigma-Aldrich | H3393-10KU | 25 µg/ml |
